# A two-phase thin-film model for cell-induced gel contraction incorporating osmotic effects

**DOI:** 10.1007/s00285-024-02072-1

**Published:** 2024-04-12

**Authors:** J. R. Reoch, Y. M. Stokes, J. E. F. Green

**Affiliations:** School of Computer and Mathematical Sciences, University of Adelaide, Adelaide, SA 5005 Australia

**Keywords:** Mathematical model, Multiphase model, Gel, Osmosis, Fluid mechanics, Thin films, Cell-extracellular matrix interactions, Tissue engineering, 76A20, 76Z99, 92-10, 92C15

## Abstract

We present a mathematical model of an experiment in which cells are cultured within a gel, which in turn floats freely within a liquid nutrient medium. Traction forces exerted by the cells on the gel cause it to contract over time, giving a measure of the strength of these forces. Building upon our previous work (Reoch et al. in J Math Biol 84(5):31, 2022), we exploit the fact that the gels used frequently have a thin geometry to obtain a reduced model for the behaviour of a thin, two-dimensional cell-seeded gel. We find that steady-state solutions of the reduced model require the cell density and volume fraction of polymer in the gel to be spatially uniform, while the gel height may vary spatially. If we further assume that all three of these variables are initially spatially uniform, this continues for all time and the thin film model can be further reduced to solving a single, non-linear ODE for gel height as a function of time. The thin film model is further investigated for both spatially-uniform and varying initial conditions, using a combination of analytical techniques and numerical simulations. We show that a number of qualitatively different behaviours are possible, depending on the composition of the gel (i.e., the chemical potentials) and the strength of the cell traction forces. However, unlike in the earlier one-dimensional model, we do not observe cases where the gel oscillates between swelling and contraction. For the case of initially uniform cell and gel density, our model predicts that the relative change in the gels’ height and length are equal, which justifies an assumption previously used in the work of Stevenson et al. (Biophys J 99(1):19–28, 2010). Conversely, however, even for non-uniform initial conditions, we do not observe cases where the length of the gel changes whilst its height remains constant, which have been reported in another model of osmotic swelling by Trinschek et al. (AIMS Mater Sci 3(3):1138–1159, 2016; Phys Rev Lett 119:078003, 2017).

## Introduction

Tissues in vivo consist of cells living within an extracellular matrix (ECM) comprising a complex network of proteins, glycoproteins, and polysaccharides that surrounds and supports cells within tissues. The ECM provides structural support, forming a scaffold that holds cells together and provides a framework for tissue organisation. It also helps to regulate cell behaviour and tissue function. For example, the ECM contains adhesive proteins, such as fibronectin and laminin, which allow cells to attach to the matrix. This adhesion is essential for cell anchoring and migration, influencing processes like wound healing and tissue regeneration. The ECM acts as a reservoir for growth factors, cytokines, and other signaling molecules. These molecules are stored within the matrix and can be released in response to specific stimuli, influencing nearby cells. It also contributes to the mechanical properties of tissues, such as elasticity, stiffness, and tensile strength (Rozario and DeSimone 2010; Frantz et al. 2010; Humphrey et al. 2014; Dolega et al. 2021). During tissue development and morphogenesis, the ECM guides cell migration, and tissue architecture and shape. It provides spatial cues that direct cell movements and influences the formation of complex tissue structures (Dyson et al. 2016). Importantly, the interactions between cells and the ECM are reciprocal: the ECM is dynamically regulated and can undergo remodeling and degradation by the cells e.g., through the secretion of enzymes called matrix metalloproteinases (MMPs). This process is crucial for tissue repair, regeneration, and remodeling after injury or during normal physiological processes.

In order to recreate a more in vivo-like cellular environment in vitro, cells are often cultured within a gel that emulates (to some degree) the ECM. Collagen, a structural protein that constitutes a major component of the ECM in many animal tissues, is commonly utilized in laboratory studies for this purpose. However, a diverse range of other natural (e.g., Matrigel) or synthetic (e.g., poly(lactic acid)) gels are also employed (Wade and Burdick 2012). Improved understanding of the mechanics of such gels, and of the interactions between cells and the gel, will yield deeper insight into tissue development and functionality. One well-known experiment used to study and quantify how cells remodel the extracellular matrix is the collagen gel contraction assay (Moon and Tranquillo 1993; Barocas et al. 1995). In this assay, cells are cultured within a gel made of collagen, which in turn floats within a bath of nutrient medium. As the cells exert force, they compress the collagen fibres, leading to a reduction in the size of the gel over a period of hours or days. Whilst other gel geometries have been used (e.g., microspheres (Moon and Tranquillo 1993)), a thin disc shape is common, due to ease of fabrication (Vernon and Gooden 2002; Stevenson et al. 2010). Whilst it is expected that cell-induced compaction leads to a reduction in both the diameter and height of the collagen disc, typically the extent of contraction is assessed by measuring only the diameter or the area of the disc (Vernon and Gooden 2002; Stevenson et al. 2010).

In an earlier paper, we developed a model for cell-induced gel compaction incorporating osmotically-driven movement of solvent in or out of the gel (Reoch et al. 2022). This model treats the gel as a two-phase fluid, consisting of polymer and solution, based on previous work (Keener et al. 2011b, a; Mori et al. 2013). However, it additionally includes an equation for the cell density, with the cells exerting a body force on the polymer; mechanical changes in the gel due to this body force were assumed to occur on a short timescale compared with that of cell proliferation and death, so that proliferation and death were neglected. This is reasonable where mechanical changes occur over hours and cell proliferation and death occurs over days. The resulting system of equations was then investigated in a one-dimensional geometry (Reoch et al. 2022). Motivated by the thin geometry commonly used in experiments, in this paper we investigate the behaviour of two-dimensional thin films of gel floating freely in a bath of solvent. We study the 2D Cartesian coordinate system for simplicity of modelling and to facilitate comparison with the 1D Cartesian model presented in earlier work. We continue to neglect cell proliferation and death, assuming the timescale of these process to be much longer than remodelling of the gel under the influence of cell forces. Our two aims are: firstly, to understand the mechanics of the gel in the thin film geometry; and secondly, to compare the emergent behaviours in this system to those in the 1D case. Once more, we hypothesise that the balance between chemical potentials and cell traction stress will be crucial in determining the equilibrium outcome for the gel. To our knowledge, such a model considering the interacting effects of cell traction and osmotic pressure in the thin film geometry has not previously been presented.

Thin film complex fluid models have been employed in a range of biological applications, including modelling cell crawling (Oliver et al. 2005; King and Oliver 2005)), pattern formation in cell aggregation (Green et al. 2017), and osmotically-driven biofilm growth (Trinschek et al. 2016, 2017). In each case, as in the prototype problem of stretching a thin film of viscous fluid studied by Howell (1996), the small aspect ratio of the film (the ratio of its vertical to horizontal length scales) is exploited to reduce the two-dimensional mass and momentum balance equations to a one-dimensional system at leading order. However, we note that, compared to single-phase fluid models, the two-phase fluid models often require additional assumptions about the relative sizes of some of the parameters in the problem, in order to obtain a one-dimensional reduction. For example, in Green et al. (2017) the parameters describing drag and cell chemotactic effects relative to viscosity were assumed to be large. The chemotaxis scaling encodes the assumption that this is the main driver of cell movement, while the drag scaling couples together the movement of cell and culture medium phases. These scalings were crucial for a non-trivial leading order model to be derived. We make similar assumptions in the model reduction presented here.

This paper is organised as follows. In Sect. 2 we present and nondimensionalise the two-dimensional governing equations and boundary conditions of our model. By exploiting the thin geometry of the film, in Sect. 3 we reduce this model to a system of one-dimensional equations for the leading-order polymer volume fraction and velocity, cell density, film height and film length. Conditions for steady state solutions are then presented in Sect.  4. In Sect. 5, we show that when the initial conditions are spatially uniform, the thin film model can be further reduced to a single ordinary differential equation for the film height as a function of time, and present numerical solutions in this case. In Sect. 7, we consider the behaviour of small spatial perturbations to the initial conditions, before undertaking numerical simulations of the full spatially-varying thin film model in Sect. 8. The paper concludes in Sect. 9 with a discussion of our main results, and suggestions for future work.

## Mathematical model

**Fig. 1:**
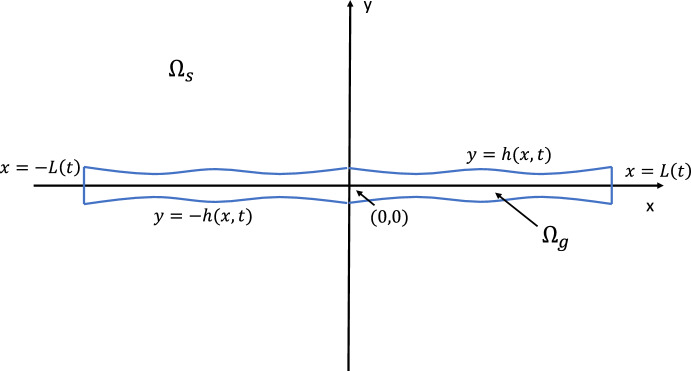
Thin film domain $\Omega = \Omega _g\cup \Omega _s$. $\Omega _g$ is the gel region with $\theta _p>0$, $\theta _s>0$ and cell density $n \ge 0$. $\Omega _s$ is the region of pure solvent surrounding the gel wherein $\theta _p= n =0$ and $\theta _s=1$. The gel is symmetric about the *x*-axis and *y*-axis, and the ratio of gel height to length is small

We consider a thin layer of gel consisting of polymer and solvent, which is seeded with cells, and floats freely within a bath of solvent. The set-up is illustrated in Fig. 1. The gel domain is denoted by $\Omega _g$, and the surrounding pure solvent domain by $\Omega _s$. We adopt a two-dimensional, Cartesian geometry, with spatial coordinates $\varvec{x} = (x, y)$. The gel is assumed to be symmetric about $y=0$ and $x=0$, with free boundaries at $y= \pm h(x,t)$ such that the gel height is 2*h*(*x*, *t*), as well as vertical free boundaries at each end $x= \pm L(t)$ giving a gel length of 2*L*(*t*). The centre-line of the gel is fixed at $y=0$. This allows us to restrict our attention to the region $0 \le y \le h$, $0 \le x \le L$. We assume that all quantities are continuous and differentiable across $x=0$ and $y=0$. Terms in the external solvent region $\Omega _s$ will henceforth be denoted with the superscript *e*; all other terms refer to quantities in the gel region $\Omega _g$. The thin gel layer is characterised by a small aspect ratio $\varepsilon$, that is, a small vertical length scale relative to the horizontal length scale (a fact we will exploit in Sect. 3).

Our mathematical model is the same as presented in Reoch et al. (2022) and Reoch (2020), though we briefly recapitulate the equations here for completeness. We let the volume fractions of the polymer and solvent be $\theta _p$ and $\theta _s$, respectively, and assume that their mass densities are equal. The cells are assumed to occupy negligible volume, and their density (number per unit volume) is denoted by *n*. (As explained in Reoch et al. (2022), this latter assumption is based on information from the experimental papers by Iordan et al. (2010) and Moon and Tranquillo (1993) which suggest the volume fraction of the cells when seeded is often *O*(0.01) or lower.) We assume there are no voids within the gel, so that

2.1 $$\begin{aligned} \theta _p+ \theta _s=1. \end{aligned}$$

The velocities of the polymer and solvent are denoted by

2.2 $$\begin{aligned} \varvec{v_p}&= (v_p, w_p), \qquad \varvec{v_s} = (v_s, w_s), \end{aligned}$$

respectively, where $v_p$ is the polymer velocity in the horizontal direction and $w_p$ is the polymer velocity in the vertical direction, and similarly for the solvent. Cells are assumed to move by advection with the polymer, and by random motion with diffusion coefficient *D*. Cell proliferation and death are taken to be negligible. Conservation of mass for the polymer, solvent and cells then gives:

2.3a $$\begin{aligned} \frac{\partial {\theta _p}}{\partial {t}} + \frac{\partial {}}{\partial {x}}(\theta _pv_p) + \frac{\partial {}}{\partial {y}}(\theta _pw_p)&= 0, \end{aligned}$$

2.3b $$\begin{aligned} \frac{\partial {\theta _s}}{\partial {t}} + \frac{\partial {}}{\partial {x}}(\theta _sv_s) + \frac{\partial {}}{\partial {y}}(\theta _sw_s)&= 0, \end{aligned}$$

2.3c $$\begin{aligned} \frac{\partial {n}}{\partial {t}} + \frac{\partial {}}{\partial {x}} (n v_p) + \frac{\partial {}}{\partial {y}}(n w_p)&= D \left( \frac{\partial ^2 n}{\partial x^2} + \frac{\partial ^2 n}{\partial y^2} \right) . \end{aligned}$$

Adding equations (2.3a) and (2.3b) and using the no-voids condition $\theta _p+ \theta _s= 1$ gives

2.4 $$\begin{aligned} \frac{\partial {}}{\partial {x}}\left( \theta _pv_p + \theta _sv_s \right) + \frac{\partial {}}{\partial {y}} \left( \theta _pw_p + \theta _sw_s \right) = 0. \end{aligned}$$

We model both the polymer and the solvent phases as fluids with a common pressure, *P*. The polymer is treated as a viscous fluid, where the viscous stress is encapsulated by the stress tensor $\varvec{\sigma }_p$, related to the rate of strain tensor $\varvec{e}_p$ by

$$\begin{aligned} \varvec{\sigma }_p = 2 \eta _p \varvec{e}_p + \kappa _p \varvec{I} \nabla \cdot \varvec{v}_p, \qquad \varvec{e}_p = \frac{1}{2} \left( \nabla \varvec{v}_p + {\nabla \varvec{v}_p} ^T \right) , \end{aligned}$$

where the constants $\eta _i$ and $\kappa _i$, $i = p,s$, are the dynamic and bulk viscosities, and $\varvec{I}$ is the identity tensor. Whilst similar assumptions are made for the solvent in Reoch et al. (2022), here, for simplicity, we take the solvent viscosities $\eta _s$ and $\kappa _s$ to be zero as in Green et al. (2017), which implies that the solvent stress tensor $\varvec{\sigma }_s = \varvec{0}$. As in Keener et al. (2011b) and Reoch et al. (2022), we assume that the forces exerted on the two phases come from inter-phase drag (which is proportional to the product of the volume fractions of the two phases), chemical potential gradients and cell-generated forces (which are exerted only on the polymer). We denote the (constant) drag coefficient by $\xi$, the chemical potentials for the polymer and solvent by $\mu _p(\theta _p)$ and $\mu _s(\theta _p)$, respectively, and the cell force potential by *G*(*n*). Conservation of momentum for the polymer and solvent phases then gives

2.5 $$\begin{aligned} \nabla \cdot (\theta _p\varvec{\sigma _p}) - \theta _p\nabla \mu _p - \theta _p\nabla P + \nabla (\theta _pG)&= \xi \theta _p\theta _s(\varvec{v_p} - \varvec{v_s}), \end{aligned}$$

2.6 $$\begin{aligned} - \theta _s\nabla \mu _s - \theta _s\nabla P&= - \xi \theta _p\theta _s(\varvec{v_p} - \varvec{v_s}). \end{aligned}$$

For the chemical potentials, we use the same forms as Keener et al. (2011b) and Reoch et al. (2022). These are:

2.7 $$\begin{aligned} \mu _p(\theta _p) = f + \theta _s\frac{\partial {f}}{\partial {\theta _p}}, \qquad \mu _s(\theta _p) = f - \theta _p\frac{\partial {f}}{\partial {\theta _p}}, \end{aligned}$$

where $f(\theta _p)$ is the free energy per unit volume of gel. In turn, the free energy function is given by

2.8 $$\begin{aligned}&f(\theta _p) = \frac{k_B {\mathcal {T}}}{\nu _m} \left( \frac{\theta _p}{N} \log (\theta _p) + \theta _s\log (\theta _s) +\chi \theta _p\theta _s+ \mu _p^0 \theta _p+ \mu _s^0 \theta _s\right) , \end{aligned}$$

where $k_B$ is the Boltzmann constant, ${\mathcal {T}}$ is temperature, $\nu _m$ is the characteristic volume of a monomer in our system, *N* is the chain length of the polymer, $\chi$ is the Flory interaction parameter and the constants $\mu _p^0$ and $\mu _s^0$ are dimensionless quantities known as the standard free energies of the polymer and solvent respectively. The logarithmic terms in the function describe the entropy of mixing polymer and solvent; these terms always encourage swelling in the gel. The latter terms involving $\chi$, $\mu _p^0$ and $\mu _s^0$ can increase the tendency for the gel to swell or contract depending on the signs of these parameters. The $\chi$ term describes the energy of mixing, while the terms involving $\mu _p^0$ and $\mu _s^0$ describe the interaction energy in a pure polymer or solvent state respectively (Rubinstein and Colby 2003).

Likewise, following Reoch et al. (2022), for the cell potential we set

2.9 $$\begin{aligned} G(n) = \frac{\tau _0 n^2}{1 + \lambda n^2}. \end{aligned}$$

The form of this function is similar to that used in earlier works, such as Murray (2001), Moon and Tranquillo (1993), but differs by having a factor of $n^2$ rather than *n* in the numerator. This ensures that $\partial {G} / \partial {n} > 0$ for all *n*, which implies that the cell traction force, $\nabla G =G'(n) \nabla n$, is directed up gradients of cell density. The parameter $\tau _0 \ge 0$ provides a measure for the strength of cell traction forces, and $\lambda \ge 0$ is a contact inhibition parameter, which reduces the force exerted by cells as they become more densely packed.

Taking the sum of equations (2.6) and (2.5) and using the relation $\theta _p\nabla \mu _p = -\theta _s\nabla \mu _s$ (which can be derived from (2.7)) we can express equation (2.5) in the form

2.10 $$\begin{aligned} \nabla \cdot (\theta _p\varvec{\sigma }_p) - \nabla P + \nabla (\theta _pG) = 0 . \end{aligned}$$

Equations (2.6) and (2.10) are used as our momentum balance equations hereafter.

### Boundary and initial conditions

Our model consists of equations (2.1), (2.3), (2.6) and (2.10), which require suitable boundary and initial conditions. These will now be specified.

Firstly, we consider the motion of the free boundaries at $y=h(x,t)$ and $x=L(t)$, which is given by the standard kinematic conditions

2.11 $$\begin{aligned} w_p&= \frac{\partial {h}}{\partial {t}} + v_p \frac{\partial {h}}{\partial {x}} \quad \hbox { at}\ y=h, \end{aligned}$$

2.12 $$\begin{aligned} v_p&= \frac{dL}{dt} \quad \hbox { at}\ x=L. \end{aligned}$$

These conditions imply that polymer ‘particles’ on the gel’s surface must always remain there. We further assume continuity of stress across these free boundaries, hence

2.13 $$\begin{aligned} \theta _p\varvec{\sigma }_p\cdot \varvec{{\hat{n}}}+[ \theta _pG - P] \varvec{{\hat{n}}}= {\varvec{0}}, \end{aligned}$$

where the square bracket notation indicates the jump across the boundary. Note that our assumption that the viscosity of the solvent is negligible implies zero tangential stress. Following Mori et al. (2013) we assume that the solvent flux across the gel boundary satisfies

2.14 $$\begin{aligned} {\mathcal {R}}\theta _s(\varvec{v}_s - \varvec{v}_p) \cdot \varvec{{\hat{n}}}= \left( \varvec{{\hat{n}}}\cdot \varvec{\sigma }_s^e \cdot \varvec{{\hat{n}}}\right) - \left( \varvec{{\hat{n}}}\cdot {\varvec{\sigma }_s} \cdot \varvec{{\hat{n}}}\right) + [P + \mu _s], \end{aligned}$$

where, as before noted, the superscript *e* indicates a quantity in the solvent domain external to the gel. This condition describes how the difference in pressure, chemical potential, and solvent stress across the interface drives fluid flow into or out from the gel, at a rate dependent on the resistance ${\mathcal {R}}\ge 0$ of the boundary (an increase in ${\mathcal {R}}$ implies the boundary is less permeable to solvent flow).

Evaluating the force balance equation (2.6) in the solvent domain $\Omega _s$, we find that the external pressure $P^{e}$ is at most a function of time, i.e. $P^{e} = P^{e}(t)$; we also note that $\mu _s^{e} = f(0)$, where *f*(0) is constant.

For the cells, the assumed symmetry of the system about $x=0$ implies

2.15 $$\begin{aligned} \frac{\partial {n}}{\partial {x}} = 0 \quad \text {at }x=0. \end{aligned}$$

We impose no-flux cell boundary conditions on $y=h$ and $x=L$, which implies

2.16 $$\begin{aligned} \nabla n \cdot \varvec{{\hat{n}}} =0, \end{aligned}$$

where $\varvec{{\hat{n}}}$ is the unit outward-pointing normal. We assume that the centre of the gel at (0, 0) is stationary. The assumed symmetries of the domain about $x=0$ and $y=0$ imply that $v_p$ must be an odd function of *x* and an even function of *y*. They similarly imply that $w_p$ is an even function of *x* and odd function of *y*. We assume that all the velocities are differentiable throughout the domain. Hence we have:

2.17 $$\begin{aligned} v_p(0, y, t)= & {} 0, \qquad \frac{\partial {v_p}}{\partial {y}}\bigg \vert _{y=0} = 0, \end{aligned}$$

2.18 $$\begin{aligned} w_p(x, 0, t)= & {} 0, \qquad \frac{\partial {w_p}}{\partial {x}}\bigg \vert _{x=0} = 0. \end{aligned}$$

Note that these conditions similarly hold for $v_s$ and $w_s$ and their derivatives, although they will not be required for solving the model equations.

Initial conditions are required for $\theta _p$, *n*, *h*, and *L*. We note that those for $\theta _p$, *n* and *h* must satisfy the assumed symmetries of the gel about $x=0$ and $y=0$. A variety of suitable functional forms are considered in subsequent sections. A table summarising the notation used in this paper is given in Appendix A.

### Nondimensionalisation and scaling

We now re-scale and nondimensionalise our system to facilitate simplification and analysis. We let ${\mathcal {L}} = L(0)$ be our length scale, ${\mathcal {N}}$ be a characteristic cell density which we choose to be the average cell density at $t=0$, and set the time scale to be the ratio of polymer viscosity $\eta _p$ to the free energy scale $k_B {\mathcal {T}} / \nu _m$. We let ${\mathcal {H}}$ be the height scale, and set this as the average height at $t=0$. Thus, the aspect ratio is defined as $\varepsilon = {\mathcal {H}} / {\mathcal {L}}$. We then nondimensionalise the independent variables as follows (where tildes denote dimensionless quantities)

$$\begin{aligned}&x = {\mathcal {L}} {\tilde{x}}, \quad y = \varepsilon {\mathcal {L}} {\tilde{y}}, \quad t = \frac{\eta _p \nu _m}{k_B {\mathcal {T}}} {\tilde{t}}, \end{aligned}$$

with the dependent variables and functions being nondimensionalised thus:

$$\begin{aligned} n= & {} {\mathcal {N}}{\tilde{n}}, \quad L(t) = {\mathcal {L}}{\tilde{L}}({\tilde{t}}), \quad h = \varepsilon {\mathcal {L}} {\tilde{h}} = {\mathcal {H}} {\tilde{h}}, \quad v_p = \frac{{\mathcal {L}} k_B {\mathcal {T}}}{\eta _p \nu _m} {\tilde{v}}_p, \quad v_s = \frac{{\mathcal {L}} k_B {\mathcal {T}}}{\eta _p \nu _m} {\tilde{v}}_s, \\ w_p= & {} \frac{\varepsilon {\mathcal {L}} k_B {\mathcal {T}}}{\eta _p \nu _m} {\tilde{w}}_p, \quad w_s = \frac{\varepsilon {\mathcal {L}} k_B {\mathcal {T}} }{\eta _p \nu _m} {\tilde{w}}_s, \quad P = \frac{k_B {\mathcal {T}}}{\varepsilon ^2 \nu _m} {\tilde{P}}, \quad \mu _s = \frac{k_B {\mathcal {T}}}{\varepsilon ^2 \nu _m} \tilde{\mu }_s. \end{aligned}$$

The forms of equations (2.1), (2.3a) and (2.3b) are unchanged upon re-scaling, whilst equation (2.3c) becomes

2.19 $$\begin{aligned} \frac{\partial {{\tilde{n}}}}{\partial {{\tilde{t}}}} + \frac{\partial {}}{\partial {{\tilde{x}}}} ({\tilde{n}} {\tilde{v}}_p) + \frac{\partial {}}{\partial {{\tilde{y}}}}({\tilde{n}} {\tilde{w}}_p)&= {\tilde{D}} \frac{\partial ^2 {\tilde{n}}}{\partial {\tilde{x}}^2} + \frac{{\tilde{D}}}{\varepsilon ^2} \frac{\partial ^2 {\tilde{n}}}{\partial {\tilde{y}}^2}, \end{aligned}$$

where we have introduced the dimensionless diffusion coefficient,

2.20 $$\begin{aligned} {\tilde{D}} = \frac{\eta _p \nu _m}{{\mathcal {L}}^2 k_B {\mathcal {T}}} D. \end{aligned}$$

The momentum balance equations (2.6) and (2.10) (in the *x* and *y* directions respectively) now take the form

2.21a $$\begin{aligned}{} & {} \theta _s\frac{\partial {\tilde{\mu }_s}}{\partial {{\tilde{x}}}} + \theta _s\frac{\partial {{\tilde{P}}}}{\partial {{\tilde{x}}}} - \tilde{\xi } \theta _p\theta _s({\tilde{v}}_p - {\tilde{v}}_s) = 0, \end{aligned}$$

2.21b $$\begin{aligned}{} & {} \theta _s\frac{\partial {\tilde{\mu }_s}}{\partial {{\tilde{y}}}} + \theta _s\frac{\partial {{\tilde{P}}}}{\partial {{\tilde{y}}}} - \varepsilon ^2 \tilde{\xi } \theta _p\theta _s({\tilde{w}}_p - {\tilde{w}}_s) = 0, \end{aligned}$$

2.22a $$\begin{aligned}{} & {} - \frac{\partial {{\tilde{P}}}}{\partial {{\tilde{x}}}} + \frac{\partial {}}{\partial {{\tilde{x}}}} \left( \theta _p\frac{\tilde{\tau }_0 {\tilde{n}}^2}{1 + \tilde{\lambda } {\tilde{n}}^2} \right) + \frac{\partial {}}{\partial {{\tilde{y}}}} \left( \theta _p\frac{\partial {{\tilde{v}}_p}}{\partial {{\tilde{y}}}} \right) \nonumber \\{} & {} \quad + \varepsilon ^2 \left\{ \frac{\partial {}}{\partial {{\tilde{x}}}} \left( 2 \theta _p\frac{\partial {{\tilde{v}}_p}}{\partial {{\tilde{x}}}} + \tilde{\kappa }_p \theta _p\left( \frac{\partial {{\tilde{v}}_p}}{\partial {{\tilde{x}}}} + \frac{\partial {{\tilde{w}}_p}}{\partial {{\tilde{y}}}}\right) \right) + \frac{\partial {}}{\partial {{\tilde{y}}}}\left( \theta _p\frac{\partial {{\tilde{w}}_p}}{\partial {{\tilde{x}}}} \right) \right\} = 0, \nonumber \\ \end{aligned}$$

2.22b $$\begin{aligned}{} & {} -\frac{1}{\varepsilon ^2}\left\{ \frac{\partial {{\tilde{P}}}}{\partial {{\tilde{y}}}} - \frac{\partial {}}{\partial {{\tilde{y}}}} \left( \theta _p\frac{\tilde{\tau }_0 {\tilde{n}}^2}{1+\tilde{\lambda }{\tilde{n}}^2} \right) \right\} +\varepsilon ^2 \left\{ \frac{\partial {}}{\partial {{\tilde{x}}}} \left( \theta _p\frac{\partial {{\tilde{w}}_p}}{\partial {{\tilde{x}}}} \right) \right\} \nonumber \\{} & {} \quad + \frac{\partial {}}{\partial {{\tilde{y}}}} \left( 2 \theta _p\frac{\partial {{\tilde{w}}_p}}{\partial {{\tilde{y}}}} + \tilde{\kappa }_p \theta _p\left( \frac{\partial {{\tilde{v}}_p}}{\partial {{\tilde{x}}}} + \frac{\partial {{\tilde{w}}_p}}{\partial {{\tilde{y}}}}\right) \right) + \frac{\partial {}}{\partial {{\tilde{x}}}}\left( \theta _p\frac{\partial {{\tilde{v}}_p}}{\partial {{\tilde{y}}}}\right) = 0, \end{aligned}$$

where we have defined the dimensionless parameters

2.23 $$\begin{aligned}&\tilde{\kappa }_p = \frac{\kappa _p}{\eta _p}, \quad \tilde{\xi } = \frac{\varepsilon ^2 {\mathcal {L}}^2 \xi }{\eta _p}, \quad \tilde{\tau }_0 = \frac{\varepsilon ^2 \nu _m {\mathcal {N}}^2 \tau _0}{k_B {\mathcal {T}}}, \quad \tilde{\lambda } = {\mathcal {N}}^2\lambda . \end{aligned}$$

Note that $\tilde{\xi }$ and $\tilde{\tau }_0$ are taken to be ${\mathcal {O}} (1)$. This reflects our assumptions that the unscaled drag and cell traction parameters are large.

We now likewise nondimensionalise the boundary conditions. We begin by noting that the kinematic boundary conditions (2.11) and (2.12) are unchanged in form after being re-scaled. On $y=h$, the stress conditions (2.13) (in the normal and tangential directions, respectively) and the solvent flux condition (2.14) now become:

2.24 $$\begin{aligned}&- \varepsilon ^2 \left\{ 2 \theta _p\frac{\partial {{\tilde{v}}_p}}{\partial {{\tilde{x}}}} + \tilde{\kappa }_p \theta _p\left( \frac{\partial {{\tilde{v}}_p}}{\partial {{\tilde{x}}}} + \frac{\partial {{\tilde{w}}_p}}{\partial {{\tilde{y}}}} \right) \right\} \frac{\partial {{\tilde{h}}}}{\partial {{\tilde{x}}}} + \varepsilon ^2 \theta _p\frac{\partial {{\tilde{w}}_p}}{\partial {{\tilde{x}}}} \nonumber \\&\quad - \left( {{\tilde{P}}_\vartriangle } - \theta _p\frac{\tilde{\tau }_0 {\tilde{n}}^2}{1 + \tilde{\lambda } {\tilde{n}}^2} \right) \frac{\partial {{\tilde{h}}}}{\partial {{\tilde{x}}}} + \theta _p\frac{\partial {{\tilde{v}}_p}}{\partial {{\tilde{y}}}} = 0, \end{aligned}$$

2.25 $$\begin{aligned}&-\varepsilon ^2 \theta _p\frac{\partial {{\tilde{w}}_p}}{\partial {{\tilde{x}}}} \frac{\partial {{\tilde{h}}}}{\partial {{\tilde{x}}}} - \theta _p\frac{\partial {{\tilde{v}}_p}}{\partial {{\tilde{y}}}} \frac{\partial {{\tilde{h}}}}{\partial {{\tilde{x}}}} + 2 \theta _p\frac{\partial {{\tilde{w}}_p}}{\partial {{\tilde{y}}}} + \tilde{\kappa }_p \theta _p\left( \frac{\partial {{\tilde{v}}_p}}{\partial {{\tilde{x}}}} + \frac{\partial {{\tilde{w}}_p}}{\partial {{\tilde{y}}}} \right) \nonumber \\&\quad - \frac{1}{\varepsilon ^2} \left( {\tilde{P}}_\vartriangle - \theta _p\frac{\tilde{\tau }_0 {\tilde{n}}^2}{1 + \tilde{\lambda }{\tilde{n}}^2} \right) = 0, \end{aligned}$$

2.26 $$\begin{aligned}&- \tilde{{\mathcal {R}}} \theta _s({\tilde{v}}_s - {\tilde{v}}_p) \frac{\partial {{\tilde{h}}}}{\partial {{\tilde{x}}}} \frac{1}{\sqrt{\varepsilon ^2 \frac{\partial {{\tilde{h}}}}{\partial {{\tilde{x}}}}^2 + 1}}+ \tilde{{\mathcal {R}}} \theta _s({\tilde{w}}_s - {\tilde{w}}_p) \frac{1}{\sqrt{\varepsilon ^2 \frac{\partial {{\tilde{h}}}}{\partial {{\tilde{x}}}}^2 + 1}}= {\tilde{P}}_\vartriangle + \tilde{\mu }_{s \vartriangle } , \end{aligned}$$

where the non-dimensional resistance parameter is defined as

2.27 $$\begin{aligned} \tilde{{\mathcal {R}}} = \frac{\varepsilon ^3 {\mathcal {L}} {\mathcal {R}}}{\eta _p}, \end{aligned}$$

and we have introduced the notation $P_\vartriangle = P - P^{e}$ and $\mu _{s_\vartriangle } = \mu _s - \mu _s^{e}$. As with the drag and cell traction parameters ($\tilde{\xi }$ and $\tilde{\tau }_0$ respectively) above, the scaled resistance parameter is taken to be ${\mathcal {O}} (1)$, indicating that resistance is significant.

At at ${{\tilde{x}}}={{\tilde{L}}}$, the stress and flux conditions (2.13) and (2.14) equivalent to those above become

2.28 $$\begin{aligned}&2\theta _p\frac{\partial {{\tilde{v}}_p}}{\partial {{\tilde{x}}}} + \tilde{\kappa }_p \theta _p\left( \frac{\partial {{\tilde{v}}_p}}{\partial {{\tilde{x}}}} + \frac{\partial {{\tilde{w}}_p}}{\partial {{\tilde{y}}}} \right) - \frac{1}{\varepsilon ^2} \left( {\tilde{P}}_\vartriangle - \theta _p\frac{\tilde{\tau }_0 {\tilde{n}}^2}{1+\tilde{\lambda }{\tilde{n}}^2} \right) = 0, \end{aligned}$$

2.29 $$\begin{aligned}&\varepsilon ^2 \theta _p\frac{\partial {{\tilde{w}}_p}}{\partial {{\tilde{x}}}} + \theta _p\frac{\partial {{\tilde{v}}_p}}{\partial {{\tilde{y}}}} = 0, \end{aligned}$$

2.30 $$\begin{aligned}&\tilde{{\mathcal {R}}} \theta _s({\tilde{v}}_s - {\tilde{v}}_p) = \varepsilon ({\tilde{P}}_\vartriangle + \tilde{\mu }_{s \vartriangle } ). \end{aligned}$$

Rescaling the no-flux boundary condition (2.16) at ${{\tilde{y}}}={{\tilde{h}}}$ gives

2.31 $$\begin{aligned} -\varepsilon ^2 {\tilde{D}} \frac{\partial {{\tilde{n}}}}{\partial {{\tilde{x}}}} \frac{\partial {{\tilde{h}}}}{\partial {{\tilde{x}}}} + {\tilde{D}} \frac{\partial {{\tilde{n}}}}{\partial {{\tilde{y}}}} = 0, \end{aligned}$$

whilst at ${{\tilde{x}}}={{\tilde{L}}}$ it becomes

2.32 $$\begin{aligned} {\tilde{D}} \frac{\partial {{\tilde{n}}}}{\partial {{\tilde{x}}}} =0. \end{aligned}$$

The symmetry conditions on the velocities, (2.17) and (2.18), retain the same forms after nondimensionalisation.

In order to simplify our notation, we henceforth drop tildes from dimensionless quantities.

## Thin film approximation

We now assume that $\varepsilon \ll 1$ and exploit this fact to obtain a simplified, one-dimensional version of our model equations. We begin by expanding our variables in powers of $\varepsilon$:

$$\begin{aligned}&\theta _p = \theta _{p_0}+ \varepsilon \theta _{p_1} + \varepsilon ^2 \theta _{p_2} +\cdots , \; n = n_0 + \varepsilon n_1 + \varepsilon ^2 n_2 + \cdots , \\&h = h_0 + \varepsilon h_1 + \varepsilon ^2 h_2 + \cdots , \end{aligned}$$

and similarly for $\theta _s$, $v_p$, $v_s$, $w_p$, $w_s$, *P* and *L*. We then substitute these expansions into our model equations. Unlike previous work discussed in Sect. 1, we expand our variables here in powers of $\varepsilon$ instead of $\varepsilon ^2$. This is due to the ${\mathcal {O}}(\varepsilon )$ term which appears in interface condition (2.30) after the model is re-scaled. As we will see, the ${\mathcal {O}}(\varepsilon )$ terms do not contribute to the leading order model we derive below, but they are included here for completeness.

### The *y*-independence of the leading order dependent variables

We begin by showing that the leading order cell density $n_0$, pressure $P_0$, polymer fraction $\theta _{p_0}$ and polymer axial velocity $v_{p_0}$ do not depend on *y*. This facilitates the derivation of simplified mass and momentum equations, as we will demonstrate subsequently.

Taking equation (2.19) at ${\mathcal {O}}(\varepsilon ^{-2})$, we find

3.1 $$\begin{aligned} D\frac{\partial ^2 n_0}{\partial y^2} = 0. \end{aligned}$$

At leading order, the no-flux boundary condition (2.31) gives $D \partial {n_0} / \partial {y} = 0$ at $y=h_0$. Integrating (3.1) and applying this no-flux boundary condition, we have

3.2 $$\begin{aligned} D\frac{\partial {n_0}}{\partial {y}} = 0, \end{aligned}$$

and so $n_0 = n_0(x,t)$. (Note that we have assumed $D \ne 0$ to derive this result.) We remark that, for our thin-film model to be consistent, this result implies we can only impose initial conditions for *n* which are independent of *y*.

Considering (2.22b) at ${\mathcal {O}}(\varepsilon ^{-2})$, we have

3.3 $$\begin{aligned} \frac{\partial {\Pi _0}}{\partial {y}} = 0, \end{aligned}$$

where we have defined $\Pi _0 = P_0 - \theta _{p_0}G_0$, noting that $G_0 = G(n_0(x,t))$. This implies that

3.4 $$\begin{aligned} \Pi _0 = \Pi _0(x,t). \end{aligned}$$

From interface condition (2.25), at $y=h_0$,

3.5 $$\begin{aligned} P_0 - {P}_0^e(t) - \theta _{p_0}{G}_0 = 0. \end{aligned}$$

We have found that ${P}_0^e(t)$ and higher order equivalent terms do not feature in the final system of equations; therefore, without loss of generality, we now set ${P}^e = 0$, and as such, $P_\vartriangle = P$ for each order of $\varepsilon$. Accordingly, applying (3.5), we find

3.6 $$\begin{aligned} \Pi _0 = 0 \quad \implies \quad P_0 = \theta _{p_0}{G}_0. \end{aligned}$$

We note that this result holds for all *y* and, accordingly, throughout the gel.

At ${\mathcal {O}}(1)$, equation (2.21b) yields

3.7 $$\begin{aligned} \theta _{s_0}\frac{\partial {}}{\partial {y}} \left( \mu _{s_0} + \theta _{p_0}{G}_0 \right)&= 0, \end{aligned}$$

where $$\mu _{s_0} = \mu _s(\theta _{p_0})$$; therefore,

3.8 $$\begin{aligned} \theta _{s_0}\left( \frac{\partial {\mu _{s_0}}}{\partial {\theta _p}} + {G}_0 \right) \frac{\partial {\theta _{p_0}}}{\partial {y}}&= 0. \end{aligned}$$

Now, for (3.8) to hold, we must have that $\partial {\theta _{p_0}} / \partial {y} = 0$ or that $\partial {\mu _{s_0}}/ \partial {\theta _p} = -{G}_0$. Given that $G_0$ is independent of *y* and that $\mu _{s_0} = \mu _s(\theta _{p_0})$, both these conditions imply that we must have

3.9 $$\begin{aligned} \theta _{p_0}= \theta _{p_0}(x,t), \end{aligned}$$

i.e. at leading order, $\theta _{p_0}$ is independent of *y*, and accordingly, $\mu _{s_0}$ is as well. Similarly, we have now found that $P_0 = P_0(x,t)$ by equation (3.6). As noted for $n_0$ above, for equation (3.9) to be true at all times, it must also hold for the initial polymer fraction $\theta _I(x,y)$, i.e. our initial condition must satisfy $\partial \theta _I(x,y) / \partial y = 0$.

Using our definition for $\Pi _0$ together with equation (2.22a) at leading order, we have

3.10 $$\begin{aligned} -\frac{\partial {\Pi _0}}{\partial {x}} + \frac{\partial {}}{\partial {y}} \left( \theta _{p_0}\frac{\partial {v_{p_0}}}{\partial {y}} \right)&= 0. \end{aligned}$$

Noting from (3.6) that $\Pi _0 = 0$, (3.10) becomes

3.11 $$\begin{aligned} \frac{\partial {}}{\partial {y}} \left( \theta _{p_0}\frac{\partial {v_{p_0}}}{\partial {y}} \right) = 0 \quad \implies \quad \theta _{p_0}\frac{\partial {v_{p_0}}}{\partial {y}} = F_1(x,t). \end{aligned}$$

From boundary condition (2.24) at ${\mathcal {O}}\left( 1 \right)$,

3.12 $$\begin{aligned} \theta _{p_0}\frac{\partial {v_{p_0}}}{\partial {y}} = 0 \hbox { at}\ y = h_0, \end{aligned}$$

and thus $F_1(x,t) = 0$. Accordingly,

3.13 $$\begin{aligned} \theta _{p_0}\frac{\partial {v_{p_0}}}{\partial {y}} = 0, \quad \implies \quad v_{p_0}= v_{p_0}(x,t), \end{aligned}$$

i.e. our leading order polymer axial velocity is independent of *y*.

From equation (2.21a) at leading order,

3.14 $$\begin{aligned} v_{s_0}= v_{p_0}-\frac{1}{\xi \theta _{p_0}} \left( \frac{\partial {\mu _{s_0}}}{\partial {x}} + \frac{\partial {}}{\partial {x}} \left( \theta _{p_0}{G}_0 \right) \right) . \end{aligned}$$

Therefore, we also have leading order solvent axial velocity $v_{s_0}= v_{s_0}(x,t)$.

We now follow the same process to show, in Appendix B, that the ${\mathcal {O}}(\varepsilon )$ terms in cell density $n_1$, pressure $P_1$, polymer fraction $\theta _{p_1}$, and polymer axial velocity $v_{p_1}$ are also independent of *y*. This simplifies later steps in our derivation of a leading-order model.

### Derivation of thin film mass balance equations

Having now established that $\theta _{p_0}$ and $v_{p_0}$ are independent of *y*, the mass conservation equation (2.3a) at leading order can be integrated with respect to *y* to give:

3.15 $$\begin{aligned} \theta _{p_0}w_{p_0}&= - \left( \frac{\partial {\theta _{p_0}}}{\partial {t}} + \frac{\partial {}}{\partial {x}} (\theta _{p_0}v_{p_0}) \right) y, \end{aligned}$$

(where we have used the fact that $w_{p_0}= 0$ at $y=0$ to determine the arbitrary function of *x* and *t* arising from the integration). The kinematic boundary condition at $y=h_0$ states that

3.16 $$\begin{aligned} w_{p_0}= \frac{\partial {h_0}}{\partial {t}} + v_{p_0}\frac{\partial {h_0}}{\partial {x}}. \end{aligned}$$

Setting $y=h_0$ in equation (3.15), and using (3.16) then yields

3.17 $$\begin{aligned} \frac{\partial {}}{\partial {t}} \left( \theta _{p_0}h_0 \right) + \frac{\partial {}}{\partial {x}} \left( \theta _{p_0}v_{p_0}h_0 \right) = 0. \end{aligned}$$

This equation describes the mass conservation of polymer over the depth of the thin film.

Similarly, integrating the solvent conservation of mass equation (2.3b) from $y=0$ to $y=h_0$, and noting that $w_{s_0}\vert _{y=0}=0$ gives

3.18 $$\begin{aligned} \theta _{s_0}w_{s_0}\bigg |_{y=h_0} = - y \left( \frac{\partial {\theta _{s_0}}}{\partial {t}} + \frac{\partial {}}{\partial {x}} \left( \theta _{s_0}v_{s_0}\right) \right) \bigg |_0^{h_0}= -h_0 \left( \frac{\partial {\theta _{s_0}}}{\partial {t}} + \frac{\partial {}}{\partial {x}} \left( \theta _{s_0}v_{s_0}\right) \right) .\nonumber \\ \end{aligned}$$

From interface condition (2.26) at leading order, we also have

3.19 $$\begin{aligned} \theta _{s_0}w_{s_0}\bigg |_{y=h_0} = \frac{1}{{\mathcal {R}}} \left( \mu _{s_0} - \mu _{s_0}^{e} + \theta _{p_0}{G}_0 \right) + \theta _{s_0}(v_{s_0}- v_{p_0}) \frac{\partial {h_0}}{\partial {x}} + \theta _{s_0}w_{p_0}\bigg |_{y=h_0}. \end{aligned}$$

Using (3.19) and the kinematic boundary condition (3.16) for $w_{p_0}$ at $y=h_0$, we obtain

3.20 $$\begin{aligned}{} & {} \frac{\partial {}}{\partial {t}} \left( \theta _{s_0}h_0 \right) + \frac{\partial {}}{\partial {x}} \left\{ \theta _{s_0}h_0 \left( v_{p_0}- \frac{1}{\xi \theta _{p_0}} \left( \frac{\partial {\mu _{s_0}}}{\partial {x}} + \frac{\partial {}}{\partial {x}}(\theta _{p_0}{G}_0) \right) \right) \right\} \nonumber \\{} & {} \quad = - \frac{1}{{\mathcal {R}}}\left( \mu _{s_0} - \mu _{s_0}^{e} + \theta _{p_0}{G}_0 \right) , \end{aligned}$$

where we have substituted for $v_{s_0}$ using equation (3.14). Equation (3.20) describes the advection of solvent within the gel. By taking linear combinations of equations (3.20) and (3.17), we can obtain the following equations for $h_0$ and $\theta _{p_0}$:

3.21 $$\begin{aligned}&\frac{\partial {h_0}}{\partial {t}} + \frac{\partial {}}{\partial {x}} \left( h_0 v_{p_0}- \frac{h_0 \theta _{s_0}}{\xi \theta _{p_0}}\left( \frac{\partial {\mu _{s_0}}}{\partial {x}} + \frac{\partial {}}{\partial {x}}(\theta _{p_0}{G}_0) \right) \right) = - \frac{1}{{\mathcal {R}}}\left( \mu _{s_0} - \mu _{s_0}^{e} + \theta _{p_0}{G}_0 \right) , \end{aligned}$$

3.22 $$\begin{aligned}&\frac{\partial {\theta _{p_0}}}{\partial {t}} + v_{p_0}\frac{\partial {\theta _{p_0}}}{\partial {x}} + \frac{\theta _{p_0}}{h_0}\frac{\partial {}}{\partial {x}} \left( \frac{h_0 \theta _{s_0}}{\xi \theta _{p_0}}\left( \frac{\partial {\mu _{s_0}}}{\partial {x}} + \frac{\partial {}}{\partial {x}}(\theta _{p_0}{G}_0) \right) \right) = \frac{\theta _{p_0}}{{\mathcal {R}}h_0}\left( \mu _{s_0} - \mu _{s_0}^e + \theta _{p_0}{G}_0 \right) . \end{aligned}$$

In order to derive an expression for $n_0$, we evaluate equation (2.19) at ${\mathcal {O}}(1)$. This gives

3.23 $$\begin{aligned} \frac{\partial {n_0}}{\partial {t}} + \frac{\partial {}}{\partial {x}}(n_0 v_{p_0}) + \frac{\partial {}}{\partial {y}}(n_0 w_{p_0}) = D \frac{\partial ^2 n_0}{\partial x^2} + D \frac{\partial ^2 n_2}{\partial y^2}, \end{aligned}$$

which we express in the following form, noting that all terms on the right-hand side are independent of *y*:

3.24 $$\begin{aligned} D \frac{\partial ^2 n_2}{\partial y^2} = \frac{\partial {n_0}}{\partial {t}} + \frac{\partial {}}{\partial {x}}(n_0 v_{p_0}) + n_0 \frac{\partial {w_{p_0}}}{\partial {y}} - D \frac{\partial ^2 n_0}{\partial x^2}. \end{aligned}$$

After integrating between $y=0$ and $y=h_0$, we have

3.25 $$\begin{aligned} D \frac{\partial {n_2}}{\partial {y}} \bigg |_0^{h_0} = \left( \frac{\partial {n_0}}{\partial {t}} + \frac{\partial {}}{\partial {x}}(n_0 v_{p_0}) + n_0 \frac{\partial {w_{p_0}}}{\partial {y}} - D \frac{\partial ^2 n_0}{\partial x^2} \right) h_0. \end{aligned}$$

We note that $\partial n_2 / \partial y = 0$ at $y=0$ from the symmetry condition (2.15). From the no-flux boundary condition (2.31) at $y=h_0$, we find that at ${\mathcal {O}} (\varepsilon ^2)$,

3.26 $$\begin{aligned} D \frac{\partial {n_2}}{\partial {y}} = D \frac{\partial {n_0}}{\partial {x}} \frac{\partial {h_0}}{\partial {x}}. \end{aligned}$$

On substituting in these expressions and rearranging, equation (3.25) becomes

3.27 $$\begin{aligned} \left( \frac{\partial {n_0}}{\partial {t}} + \frac{\partial {}}{\partial {x}}(n_0 v_{p_0}) + n_0 \frac{\partial {w_{p_0}}}{\partial {y}} \right) h_0 - D \frac{\partial {}}{\partial {x}} \left( h_0 \frac{\partial {n_0}}{\partial {x}} \right) = 0. \end{aligned}$$

Given that $h_0 > 0$ in the gel, we divide by $h_0$ and use (3.15) to substitute for $w_{p_0}$, obtaining the mass conservation equation for $n_0$,

3.28 $$\begin{aligned} \frac{\partial {n_0}}{\partial {t}} + \frac{\partial {}}{\partial {x}}(n_0 v_{p_0}) - \frac{n_0}{\theta _{p_0}} \frac{\partial {\theta _{p_0}}}{\partial {t}} - \frac{n_0}{\theta _{p_0}} \frac{\partial {}}{\partial {x}} \left( \theta _{p_0}v_{p_0}\right) - \frac{D}{h_0} \frac{\partial {}}{\partial {x}} \left( h_0 \frac{\partial {n_0}}{\partial {x}} \right) = 0. \end{aligned}$$

### Derivation of an equation for $v_{p_0}$

Having derived equations for the polymer volume fraction, cell density and the height of the film, we now close our system of leading-order thin-film equations by finding an expression for $v_{p_0}$.

At ${\mathcal {O}} (\varepsilon ^2)$ equation (2.22a) gives

3.29 $$\begin{aligned}{} & {} \frac{\partial {}}{\partial {x}}\left( 2 \theta _{p_0}\frac{\partial {v_{p_0}}}{\partial {x}} + \kappa _p \theta _{p_0}\left( \frac{\partial {v_{p_0}}}{\partial {x}} + \frac{\partial {w_{p_0}}}{\partial {y}} \right) \right) - \frac{\partial {\Pi _2}}{\partial {x}} \nonumber \\{} & {} \quad + \frac{\partial {}}{\partial {y}}\left( \theta _{p_0}\frac{\partial {w_{p_0}}}{\partial {x}}\right) + \frac{\partial {}}{\partial {y}}\left( \theta _{p_0}\frac{\partial {v_{p_2}}}{\partial {y}} \right) = 0, \end{aligned}$$

where $$\Pi _2 = P_2 - \theta _{p_0}{G}_2 - \theta _{p_2}{G}_0 - \theta _{p_1}G_1$$.

Next, we integrate equation (3.29) with respect to *y* from $y=0$ to $y=h_0$ to obtain

3.30 $$\begin{aligned}{} & {} h_0 \frac{\partial {}}{\partial {x}}\left( 2\theta _{p_0}\frac{\partial {v_{p_0}}}{\partial {x}} \right) + h_0 \frac{\partial {}}{\partial {x}} \left( \kappa _p \theta _{p_0}\frac{\partial {v_{p_0}}}{\partial {x}} \right) + \int _{0}^{h_0} \left( \frac{\partial {}}{\partial {x}} \left( \kappa _p\theta _{p_0}\frac{\partial {w_{p_0}}}{\partial {y}} - \Pi _2 \right) \right) dy \nonumber \\{} & {} \quad = -\left[ \theta _{p_0}\frac{\partial {w_{p_0}}}{\partial {x}} + \theta _{p_0}\frac{\partial {v_{p_2}}}{\partial {y}} \right] _{0}^{h_0}. \end{aligned}$$

At $y=h_0$, the boundary condition (2.24) at ${\mathcal {O}}\left( \epsilon ^2 \right)$ supplies

3.31 $$\begin{aligned}&\theta _{p_0}\frac{\partial {w_{p_0}}}{\partial {x}} + \theta _{p_0}\frac{\partial {v_{p_2}}}{\partial {y}} = \left( 2 \theta _{p_0}\frac{\partial {v_{p_0}}}{\partial {x}} + \kappa _p \theta _{p_0}\left( \frac{\partial {v_{p_0}}}{\partial {x}} + \frac{\partial {w_{p_0}}}{\partial {y}} \right) - \Pi _2 \right) \frac{\partial {h_0}}{\partial {x}}. \end{aligned}$$

Further, by symmetry at $y=0$, we have $w_{p_0}(x,0,t) = 0$, and so $\partial w_{p_0}/ \partial x = 0$ at $y=0$. We also have that $v_{p_2}$ is an even function of *y* about $y=0$, and thus $\partial v_{p_2}/ \partial y = 0$ at $y=0$. Therefore, from equation (3.30),

3.32 $$\begin{aligned}&h_0 \frac{\partial {}}{\partial {x}}\left( 2\theta _{p_0}\frac{\partial {v_{p_0}}}{\partial {x}} \right) + h_0 \frac{\partial {}}{\partial {x}} \left( \kappa _p \theta _{p_0}\frac{\partial {v_{p_0}}}{\partial {x}} \right) + \int _{0}^{h_0} \frac{\partial {}}{\partial {x}} \left( \kappa _p\theta _{p_0}\frac{\partial {w_{p_0}}}{\partial {y}} - \Pi _2 \right) dy \nonumber \\&\qquad = -\left( 2 \theta _{p_0}\frac{\partial {v_{p_0}}}{\partial {x}} + \kappa _p \theta _{p_0}\left( \frac{\partial {v_{p_0}}}{\partial {x}} + \frac{\partial {w_{p_0}}}{\partial {y}} \bigg |_{y=h_0} \right) - {\Pi _2}\bigg |_{y=h_0} \right) \frac{\partial {h_0}}{\partial {x}}. \end{aligned}$$

Applying Leibniz’ integral rule in equation (3.32), after a little algebra we obtain

3.33 $$\begin{aligned}&\frac{\partial {}}{\partial {x}}\left( 2 \theta _{p_0}\frac{\partial {v_{p_0}}}{\partial {x}} h_0 + \kappa _p \theta _{p_0}\frac{\partial {v_{p_0}}}{\partial {x}} h_0 \right) + \frac{\partial {}}{\partial {x}} \int _{0}^{h_0} \left( \kappa _p \theta _{p_0}\frac{\partial {w_{p_0}}}{\partial {y}} - \Pi _2 \right) dy = 0. \end{aligned}$$

Our next step is to eliminate the $\Pi _2$ term in (3.33). To do this, we consider (2.22b) at ${\mathcal {O}}(1)$, finding

3.34 $$\begin{aligned} \frac{\partial {}}{\partial {y}}\left( 2\theta _{p_0}\frac{\partial {w_{p_0}}}{\partial {y}} + \kappa _p \theta _{p_0}\frac{\partial {v_{p_0}}}{\partial {x}} + \kappa _p \theta _{p_0}\frac{\partial {w_{p_0}}}{\partial {y}} \right) -\frac{\partial {\Pi _2}}{\partial {y}} = 0, \nonumber \\ \implies 2\theta _{p_0}\frac{\partial {w_{p_0}}}{\partial {y}} + \kappa _p \theta _{p_0}\frac{\partial {v_{p_0}}}{\partial {x}} + \kappa _p \theta _{p_0}\frac{\partial {w_{p_0}}}{\partial {y}} - \Pi _2 = F_3(x,t), \end{aligned}$$

where $F_3(x, t)$ is an arbitrary function. At ${\mathcal {O}}(1)$, boundary condition (2.25) on $y=h_0$ states that

3.35 $$\begin{aligned} 2\theta _{p_0}\frac{\partial {w_{p_0}}}{\partial {y}} + \kappa _p \theta _{p_0}\frac{\partial {v_{p_0}}}{\partial {x}} + \kappa _p \theta _{p_0}\frac{\partial {w_{p_0}}}{\partial {y}} - \Pi _2 = 0. \end{aligned}$$

Therefore, we find $F_3(x,t) = 0$, and from (3.34),

3.36 $$\begin{aligned} \Pi _2&= (2+ \kappa _p)\theta _{p_0}\frac{\partial {w_{p_0}}}{\partial {y}} + \kappa _p \theta _{p_0}\frac{\partial {v_{p_0}}}{\partial {x}}. \end{aligned}$$

We can now simplify the integral term in (3.33). After substituting in the expression for $\Pi _2$ above and noting that the resulting integrand is independent of *y*, equation (3.33) becomes

3.37 $$\begin{aligned}&\frac{\partial {}}{\partial {x}} \left( 2 \theta _{p_0}\frac{\partial {v_{p_0}}}{\partial {x}} h_0 - 2 \theta _{p_0}\frac{\partial {w_{p_0}}}{\partial {y}} h_0 \right) = 0. \end{aligned}$$

Now, using equation (3.15) to substitute for the $w_{p_0}$ term, and integrating with respect to *x*, we find

3.38 $$\begin{aligned} 2h_0 \left( 2\theta _{p_0}\frac{\partial {v_{p_0}}}{\partial {x}} + \frac{\partial {\theta _{p_0}}}{\partial {t}} + v_{p_0}\frac{\partial {\theta _{p_0}}}{\partial {x}} \right) = F_4(y,t). \end{aligned}$$

Since the left-hand side of this expression is independent of *y*, we must have $F_4 = F_4(t)$. On substituting from equations (3.15) and (3.36) into equation (3.38) we obtain

3.39 $$\begin{aligned} h_0 \left( 2 \theta _{p_0}\frac{\partial {v_{p_0}}}{\partial {x}} - \Pi _2 + \kappa _p \theta _{p_0}\left( \frac{\partial {v_{p_0}}}{\partial {x}} + \frac{\partial {w_{p_0}}}{\partial {y}} \right) \right) = F_4(t). \end{aligned}$$

The interface condition (2.28) at $x=L_0$ then supplies

3.40 $$\begin{aligned} 2 \theta _{p_0}\frac{\partial {v_{p_0}}}{\partial {x}} + \kappa _p \theta _{p_0}\left( \frac{\partial {v_{p_0}}}{\partial {x}} + \frac{\partial {w_{p_0}}}{\partial {y}} \right) - \Pi _2 = 0. \end{aligned}$$

Thus, we find $F_4 = 0$, and equation (3.38) simplifies to

3.41 $$\begin{aligned} 2 \theta _{p_0}\frac{\partial {v_{p_0}}}{\partial {x}} + \frac{\partial {\theta _{p_0}}}{\partial {t}} + v_{p_0}\frac{\partial {\theta _{p_0}}}{\partial {x}} = 0, \end{aligned}$$

which gives a leading order expression for the polymer axial velocity $v_{p_0}$. We have thus obtained a closed system of equations for $h_0$, $\theta _{p_0}$, $n_0$ and $v_{p_0}$ - namely, (3.21), (3.22), (3.28) and (3.41) - which constitutes our simplified thin-film model.

Finally, we note from the leading order interface condition (2.30) at $x=L_0$ that $v_{s_0}= v_{p_0}$. Substituting this into (3.14), we find, at $x=L_0$,

3.42 $$\begin{aligned} \frac{\partial {\mu _{s_0}}}{\partial {x}} = -\frac{\partial {}}{\partial {x}} \left( \theta _{p_0}{G}_0 \right) . \end{aligned}$$

We also have no cell flux at $x=L_0$, therefore $\partial {n_0}/ \partial {x} = 0$, and we can express equation (3.42) as

3.43 $$\begin{aligned} \left( \frac{\partial {\mu _{s_0}}}{\partial {\theta _{p_0}}} + {G}_0 \right) \frac{\partial {\theta _{p_0}}}{\partial {x}} = 0; \end{aligned}$$

this indicates that at $x=L_0$ we must have $\partial \theta _{p_0}/ \partial x =0$. For consistency, we will always use initial conditions such that this boundary condition is satisfied. We note that physically this may not always be true; this may lead to more complicated scenarios (such as the existence of boundary layers) which we do not address here.

### Summary of thin film model equations

Given the length of the calculations in the preceding section, for convenience we now summarise the system of thin film model equations which we consider in the remaining sections of this paper. This comprises (3.22) for $\theta _p$, (3.21) for *h*, (3.28) for *n* and (3.41) for $v_p$. (Note that henceforth, we drop the zero subscript denoting leading order quantities to reduce notational clutter.) We eliminate $\partial \theta _{p_0}/ \partial t$ from equation (3.41) using (3.22), and after a little algebra, our system becomes:

3.44a $$\begin{aligned}&\frac{\partial {\theta _p}}{\partial {t}} + \frac{\partial {}}{\partial {x}} \left( \theta _pv_p \right) + \theta _p\frac{\partial {v_p}}{\partial {x}} = 0, \end{aligned}$$

3.44b $$\begin{aligned}&\frac{\partial {h}}{\partial {t}} + \frac{\partial {}}{\partial {x}} \left( h v_p \right) - 2 h \frac{\partial {v_p}}{\partial {x}} = 0,\end{aligned}$$

3.44c $$\begin{aligned}&\frac{\partial {n}}{\partial {t}} + \frac{\partial {}}{\partial {x}} \left( n v_p \right) + n\frac{\partial {v_p}}{\partial {x}} - \frac{D}{ h} \frac{\partial {}}{\partial {x}} \left( h \frac{\partial {n}}{\partial {x}} \right) = 0,\end{aligned}$$

3.44d $$\begin{aligned}&2h\frac{\partial {v_p}}{\partial {x}} - \frac{\partial {}}{\partial {x}}\left\{ \frac{\theta _sh}{\xi \theta _p} \left( \frac{\partial {\mu _{s}}}{\partial {x}} + \frac{\partial {}}{\partial {x}}\left( \theta _pG \right) \right) \right\} + \frac{1}{{\mathcal {R}}} \left( \mu _{s} - \mu _{s}^e + \theta _pG \right) = 0. \end{aligned}$$

The above system is subject to the boundary conditions

3.45a $$\begin{aligned} \frac{\partial {\theta _p}}{\partial {x}}&= 0 \quad \hbox { at}\ x=0, \, L(t), \end{aligned}$$

3.45b $$\begin{aligned} \frac{\partial {n}}{\partial {x}}&= 0 \quad \text { at }x=0, \, L(t),\end{aligned}$$

3.45c $$\begin{aligned} \frac{\partial {h}}{\partial {x}}&= 0 \quad \text { at } x=0, \end{aligned}$$

3.45d $$\begin{aligned} v_p&= 0 \quad \hbox { at}\ x=0, \end{aligned}$$

3.45e $$\begin{aligned} \overset{\varvec{{.}}}{L}&= v_p\vert _{x=L(t)}, \end{aligned}$$

and initial conditions

3.46 $$\begin{aligned} \theta _p(x, 0) = \theta _I(x), \quad n(x,0) = n_I(x), \quad h(x,0) = h_I(x), \quad L(0) = 1. \end{aligned}$$

Note that we take the initial condition $\theta _I(x)$ to be differentiable and to satisfy $\partial \theta _I(0) / \partial x = 0$, so that for all time, $\partial \theta _p/ \partial x\vert _{x=0} =0$. We similarly take the initial height $h_I(x)$ to be differentiable and satisfy $\partial h_I /\partial x \vert _{x=0} = 0$, so that $\partial h / \partial x \vert _{x=0} =0$ for all time (required by the symmetry of the problem). Finally, we note that equation (3.43) implies $\partial \theta _p/ \partial x \vert _{x=L(t)}=0$, so our initial conditions must satisfy:

3.47 $$\begin{aligned} \frac{\partial {\theta _I}}{\partial {x}} \bigg \vert _{x=0} = \frac{\partial {\theta _I}}{\partial {x}}\bigg \vert _{x=L(t)} = 0, \qquad \frac{\partial {h_I}}{\partial {x}}\bigg \vert _{x=0} = 0. \end{aligned}$$

## Steady state conditions

We now consider the steady state solutions of our thin film model (3.44). We being by returning briefly to the conservative form of the polymer advection equation (3.17). At equilibrium, this supplies

4.1 $$\begin{aligned} \frac{\partial {}}{\partial {x}} \left( \theta _ph v_p \right) = 0, \quad \Rightarrow \quad \theta _ph v_p = 0, \end{aligned}$$

where we have integrated with respect to *x* and applied the zero velocity boundary condition at $x=0$. Given $\theta _p>0$ and $h>0$ within the gel, this means that we must have $v_p =0$ at equilibrium. Since ${\dot{L}} = v_p|_{x=L(t)}$, we have $L=L^*$ (a constant). Then, from equation (3.14), we have

4.2 $$\begin{aligned} v_s = -\frac{1}{\xi \theta _p} \frac{\partial {}}{\partial {x}} \left( \mu _s + \theta _pG \right) . \end{aligned}$$

We note that since $v_p=0$, and $v_s=0$ at $x=0$ by symmetry, we require $v_s=0$ throughout the gel, as there will otherwise be motion of the solution relative to the polymer, and $\theta _p$ will vary in time. Hence, the right-hand side of (4.2) must be zero at a steady state. Using these pieces of information, equation (6.1d) implies that we must have

4.3 $$\begin{aligned} \mu _s - \mu _s^e + \theta _pG =0. \end{aligned}$$

We now turn to the cell advection–diffusion equation (6.1c), which reduces to

4.4 $$\begin{aligned} \frac{D}{h} \frac{\partial {}}{\partial {x}} \left( h \frac{\partial {n}}{\partial {x}} \right) = 0. \end{aligned}$$

Assuming $D > 0$, integrating (4.4) and applying the no-flux boundary condition (6.2b), we find,

4.5 $$\begin{aligned} h \frac{\partial {n}}{\partial {x}} =0; \end{aligned}$$

given $h>0$, this indicates that, at equilibrium,

4.6 $$\begin{aligned} \frac{\partial {n}}{\partial {x}} = 0, \end{aligned}$$

and accordingly, *n* must be spatially uniform.

Using the fact that $G=G(n)$, equation (4.3) now gives

4.7 $$\begin{aligned} \frac{\partial {}}{\partial {x}} \left( \mu _s + \theta _pG \right) = \left( \frac{\partial {\mu _s}}{\partial {\theta _p}} + G \right) \frac{\partial {\theta _p}}{\partial {x}} =0, \end{aligned}$$

and therefore, we must have

4.8 $$\begin{aligned} \frac{\partial {\theta _p}}{\partial {x}} = 0. \end{aligned}$$

Therefore, necessary and sufficient conditions for equilibrium in the thin film are spatially uniform values $\theta _p= \theta ^*$ and $n=n^*$ that satisfy (4.3). We note that there are no restrictions on *h*, i.e. the height can be non-uniform in space at the gel’s steady state. The equilibrium conditions (4.3) match those for a one-dimensional gel with $D \ne 0$, as presented in (Reoch et al. 2022). Therefore, any equilibrium solution for $\theta _p$ and *n* in the 1D Cartesian model with $D > 0$, is an equilibrium of the thin film model.

## Reduced model for uniform initial conditions

Given spatially uniform initial conditions for $\theta _p$, *n* and *h*, we now show that we can simplify the thin film system of equations (3.44) and boundary conditions (3.45) to an even simpler form. In this case, our model reduces to an ODE for *h* as a function of time, with the other variables specified in terms of *h*.

We begin by setting the initial conditions to be

5.1 $$\begin{aligned} h\vert _{t=0} = 1, \quad \theta _p\vert _{t=0} = \theta _I, \quad n\vert _{t=0} = 1, \quad L\vert _{t=0} = 1, \end{aligned}$$

where we have scaled *h* and *n* on the initial height and cell density respectively. Noting that for uniform initial conditions, the free energy and cell force are also initially uniform in *x*, this means that the velocity equation (6.1d) at initial time is

5.2 $$\begin{aligned} \frac{\partial {v_p}}{\partial {x}} \bigg \vert _{t=0} = -\frac{1}{2 {\mathcal {R}}h} \left( \mu _{s} - \mu _{s}^e + \theta _pG \right) . \end{aligned}$$

Given that the terms on the right hand side are all initially independent of *x*, the initial velocity is

5.3 $$\begin{aligned} v_p \vert _{t=0} = -\frac{1}{2 {\mathcal {R}}h} \left( \mu _{s} - \mu _{s}^e + \theta _pG \right) x, \end{aligned}$$

where we have used the condition $v_p = 0$ at $x=0$. We can then verify from equations (3.44a), (3.44b) and (3.44c) that $\theta _p$, *h* and *n* are initially independent of *x*. By making the ansatz that $v_p$ is linear in *x*, and $\theta _p$, *h* and *n* are functions of time only, on substituting into (3.44a), (3.44b) and (3.44c) we arrive at the system of ODEs:

5.4a $$\begin{aligned} \frac{d h}{d t}&= -\frac{1}{2 {\mathcal {R}}} \left( \mu _{s} - \mu _{s}^e + \theta _pG \right) , \end{aligned}$$

5.4b $$\begin{aligned} v_p(x, t)&= -\frac{1}{2 {\mathcal {R}}h} \left( \mu _{s} - \mu _{s}^e + \theta _pG \right) x = \frac{x}{h} \frac{d h}{d t}, \end{aligned}$$

5.4c $$\begin{aligned} \frac{d \theta _p}{d t}&= \frac{\theta _p}{{\mathcal {R}}h} \left( \mu _{s} - \mu _{s}^e + \theta _pG \right) = - \frac{2 \theta _p}{h} \frac{d h}{dt} ,\end{aligned}$$

5.4d $$\begin{aligned} \frac{d n}{d t}&= \frac{n}{{\mathcal {R}}h} \left( \mu _{s} - \mu _{s}^e + \theta _pG \right) = - \frac{2 n}{h} \frac{d h}{dt}. \end{aligned}$$

The kinematic condition gives

5.5 $$\begin{aligned} \frac{d L}{dt} = v_p \vert _{x=L} = \frac{L }{h} \frac{d h}{d t}. \end{aligned}$$

On applying the initial conditions, we can find $\theta _p$, $v_p$, *n* and *L* in terms of *h* as:

5.6a $$\begin{aligned} \theta _p(t)&= \frac{\theta _I }{h^2}, \end{aligned}$$

5.6b $$\begin{aligned} n(t)&= \frac{1}{h^2}, \end{aligned}$$

5.6c $$\begin{aligned} L(t)&= {h}, \end{aligned}$$

5.6d $$\begin{aligned} v_p(x,t)&= \frac{x}{h} \frac{dh}{dt}. \end{aligned}$$

Having found solutions $\theta _p$ and *n* as functions of *h*, we can also express the chemical potential $\mu _s(\theta _p)$ and cell force function *G*(*n*) as functions of *h*, and accordingly, reduce our model to the following single ODE for *h*(*T*),

5.7 $$\begin{aligned} \frac{dh}{dt} = -\frac{1}{2 {\mathcal {R}}} \left( \mu _{s}(\theta _p) - \mu _{s}^e + \theta _pG(n) \right) , \end{aligned}$$

From this reduced model, we can draw a number of conclusions about how the gel behaves under spatially uniform initial conditions. Firstly, that if there is no initial spatial variation in the volume fractions, cell density and gel height, the gel will remain uniform in space for all time. We see that the length of the gel is equal to its height as it evolves, therefore any time-variation in the free boundaries of the gel will occur in the same manner both length-wise and height-wise. We also see that the polymer fraction and cell density are inversely proportional to the squared height, scaled by the initial conditions. Finally, the reduced model is independent of drag; with no dependence on the *x*-coordinate, there is no relative motion between the polymer and solvent as the gel evolves and so no shearing takes place.

The entire system is driven by the balance between the solvent chemical potential $\mu _s$ and the cell potential $\theta _pG$ inside the gel with the external chemical potential $\mu _s^e$. If these forces are in balance, the system is in equilibrium, as expected. For the height to be increasing, and accordingly, the gel to be swelling, we require that $\mu _s^e$ is greater than the sum of $\theta _pG$ and $\mu _s$; the opposite holds for the gel to contract. The gel equilibrates when these forces are in balance, that is

5.8 $$\begin{aligned} \mu _s(\theta _p) - \mu _s^e + \theta _pG(n) = 0, \end{aligned}$$

the same condition as derived in Sect. 4 above.

The rate at which the gel evolves is determined by equation (5.7). Accordingly, increasing the resistance of the interface ${\mathcal {R}}$ slows the rate of change of *h* and the rest of the system. Similarly, larger values of parameters such as the mixing energy $\chi$ and cell traction $\tau _0$ appearing in $\mu _s$ and *G* respectively will increase the rate of gel evolution.

### Numerical simulations of the reduced model

**Fig. 2:**
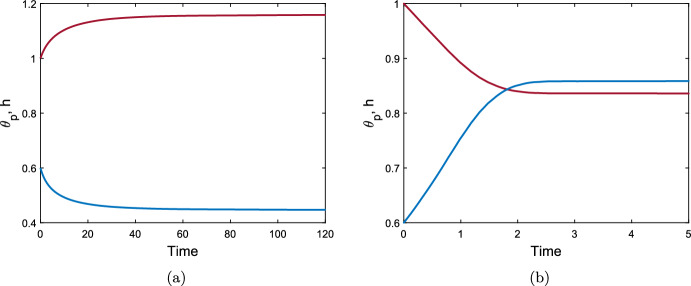
Time evolution of a cell-free thin gel with uniform initial conditions; $\theta _p$ is shown in blue, and *h* in maroon, with $h=L$. Common parameters $\theta _I = 0.6$, $n_I = 0$, $h_I = 1$, $N=100$, ${\mathcal {R}}= 1$. With $\chi = 0.75$ (Fig. 2a) the gel swells uniformly across its domain to the equilibrium ($\theta ^*, h^*, L^*) = (0.45, 1.16, 1.16)$. With $\chi =1.5$ (Fig. 2b) the gel contracts to the equilibrium (${\theta }^*, {h}^*, L^*) = (0.86, 0.84, 0.84)$

We solve the reduced model described by equations (5.6) and (5.7) using the inbuilt MATLAB ODE solver *ode15s* which is designed to handle stiff systems. For a gel without cells, we can see either swelling or contraction take place, depending on the chemical potentials in the system. Figure 2a demonstrates swelling induced by osmotic pressure (with mixing parameter $\chi = 0.75$), while, conversely, Fig. 2b shows a case where the gel contracts (with mixing increased to $\chi =1.5$, promoting separation between the polymer and solvent). The equilibria reached here, $\theta ^* = 0.45$ for the swelling case and $\theta ^* = 0.86$ for the contracting case, are, of course, the same as found for the equivalent initial conditions and parameters in the 1D Cartesian version of the model (Reoch et al. 2022; Reoch 2020).

**Fig. 3:**
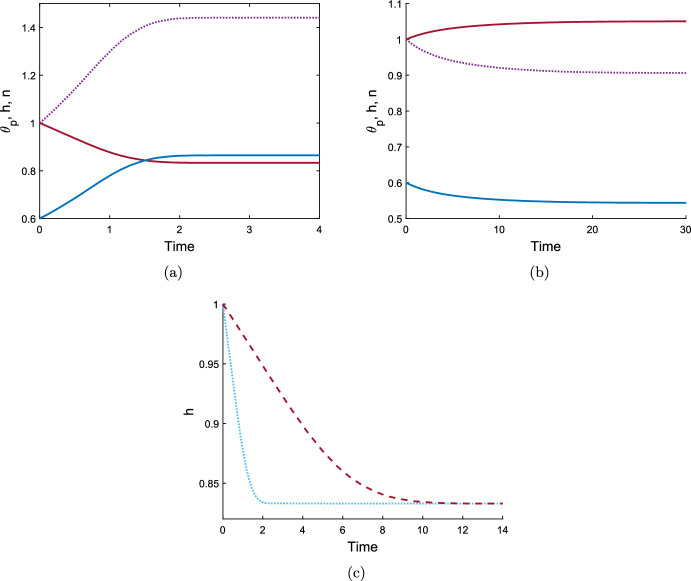
Time evolution of a thin cell-gel system with uniform initial conditions. In Fig. 3a and b, $\theta _p$ is shown by the solid blue curve, $h=L$ is the solid maroon line, and *n* is the dotted purple line, for common parameter values $\theta _I = 0.6$, $n_I = 1$, $h_I = 1$, $\chi = 0.75$, $N=100$, ${\mathcal {R}}= 1$, $\lambda =1$. With $\tau _0 = 1$ (Fig. 3a) the gel contracts to the equilibrium (${\theta }^*, n^*, {h}^*, L^*) = (0.86, 1.44, 0.83, 0.83)$ due to the presence of cells. With $\tau _0 = 0.1$ (Fig. 3b) the gel swells to a steady state due to osmoticpressure counteracting weak cell traction, (${\theta }^*, n^*, {h}^*, L^*) = (0.54, 0.91, 1.05, 1.05)$. Figure 3c compares the effect of the size of the resistance parameter on the evolution of the gel height; ${\mathcal {R}}= 1$ is shown by the light blue dotted line, ${\mathcal {R}}= 5$ by the maroon dashed line, parameter values otherwise as for Fig. 3a

Introducing cells into this system can precipitate a switch to contraction in a gel that would otherwise swell. Note that this is due to the traction forces exerted by the cells; the spatial uniformity of the cell population means that diffusion plays no role in the evolution of the system. We take the same parameter values as in the swelling gel above (Fig. 2a) and introduce a cell population ($n_I = 1$, $\tau _0=1$). In Fig. 3a, we see that this gel now contracts due to the presence of cells, reaching a steady state with a significant reduction in height and length. This highlights that in this thin film geometry, given sufficient traction stress ($\tau _0=1$), the presence of cells can outweigh the osmotic swelling pressure created by chemical potentials in a gel. Reducing the traction parameter to $\tau _0 = 0.1$ (Fig. 3b), we see that the presence of cells does not necessitate contraction; rather it is evident that, due to a weak cell contribution, osmotic pressure is still the dominant driver of the gel’s behaviour, and the thin film still swells to an equilibrium. The equilibrium values of polymer and cell density again match those found for the 1D case for the same initial conditions and parameters (Reoch et al. 2022; Reoch 2020).

Increasing the resistance parameter ${\mathcal {R}}$ slows the evolution of the gel and hence increases the time taken to reach a steady state; however, it does not affect the eventual equilibrium reached. Figure 3c shows the effect of increasing the resistance parameter from ${\mathcal {R}}= 1$ to ${\mathcal {R}}= 5$, which increases the time taken for the gel to reach its steady state approximately five-fold, from $T \approx 2$ to $T \approx 10$. This relation between ${\mathcal {R}}$ and *T* is expected, given that the solution for *h* in equation (5.7) is of the form *F*(*T*/*R*).

For both the contracting and expanding examples here, the gel remains spatially uniform throughout its evolution to steady state. The height and length are necessarily equal by (5.6c), indicating that the gel grows in a uniform ratio horizontally and vertically. Next, we will explore whether this remains the case when the gel is constructed with non-uniform initial conditions.

## Transformation to a 1D fixed domain

In order to facilitate the analysis and numerical solution of our model in spatially-varying cases, we transform to a fixed domain using the coordinate transformation $t = T$, $x = L_0(T)X$. The equations (3.44) then become:

6.1a $$\begin{aligned}&\frac{\partial {\theta _p}}{\partial {T}} - X \frac{\overset{\varvec{{.}}}{L}}{L} \frac{\partial {\theta _p}}{\partial {X}} + \frac{1}{L} \frac{\partial {}}{\partial {X}} \left( \theta _pv_p \right) + \frac{\theta _p}{L} \frac{\partial {v_p}}{\partial {X}} = 0, \end{aligned}$$

6.1b $$\begin{aligned}&\frac{\partial {h}}{\partial {T}} - X \frac{\overset{\varvec{{.}}}{L}}{L} \frac{\partial {h}}{\partial {X}} + \frac{1}{L} \frac{\partial {}}{\partial {X}} \left( h v_p \right) - \frac{2 h}{L} \frac{\partial {v_p}}{\partial {X}} = 0,\end{aligned}$$

6.1c $$\begin{aligned}&\frac{\partial {n}}{\partial {T}} - X \frac{\overset{\varvec{{.}}}{L}}{L} \frac{\partial {n}}{\partial {X}} + \frac{1}{L} \frac{\partial {}}{\partial {X}} \left( n v_p \right) + \frac{n}{L}\frac{\partial {v_p}}{\partial {X}} - \frac{D}{L^2 h} \frac{\partial {}}{\partial {X}} \left( h \frac{\partial {n}}{\partial {X}} \right) = 0,\end{aligned}$$

6.1d $$\begin{aligned}&\frac{2h}{L}\frac{\partial {v_p}}{\partial {X}} - \frac{1}{L^2}\frac{\partial {}}{\partial {X}}\left\{ \frac{\theta _sh}{\xi \theta _p} \left( \frac{\partial {\mu _{s}}}{\partial {X}} + \frac{\partial {}}{\partial {X}}\left( \theta _pG \right) \right) \right\} + \frac{1}{{\mathcal {R}}} \left( \mu _{s} - \mu _{s}^e + \theta _pG \right) = 0. \end{aligned}$$

subject to boundary conditions

6.2a $$\begin{aligned} \frac{\partial {\theta _{p_0}}}{\partial {X}}&= 0 \quad \hbox { at}\ X=0, \, 1, \end{aligned}$$

6.2b $$\begin{aligned} \frac{\partial {n_0}}{\partial {X}}&= 0 \quad \text { at }X=0, \, 1,\end{aligned}$$

6.2c $$\begin{aligned} \frac{\partial {h_0}}{\partial {X}}&= 0 \quad \hbox { at}\ X=0, \end{aligned}$$

6.2d $$\begin{aligned} v_{p_0}&= 0 \quad \hbox { at}\ X=0, \end{aligned}$$

6.2e $$\begin{aligned} \overset{\varvec{{.}}}{L}_0&= v_{p_0}\vert _{X=1}, \end{aligned}$$

and initial conditions

6.3 $$\begin{aligned} \theta _p(X, 0) = \theta _I(X), \quad n(X,0) = n_I(X), \quad h(X,0) = h_I(X), \quad L(0) = 1. \end{aligned}$$

Note that our assumptions regarding the symmetries, etc. of the initial conditions (3.47) now become:

6.4 $$\begin{aligned} \frac{\partial {\theta _I}}{\partial {X}} \bigg \vert _{X=0} = \frac{\partial {\theta _I}}{\partial {X}}\bigg \vert _{X=1} = 0, \qquad \frac{\partial {h_I}}{\partial {X}}\bigg \vert _{X=0} = 0. \end{aligned}$$

## Small time evolution of spatially perturbed uniform equilibria

We begin our investigation of the behaviour of the model with non-uniform initial conditions by considering the short time behaviour when equilibrium initial conditions are subject to spatial perturbations. This analysis will provide insight into the stability of equilibria: unstable equilibria will see an increase in the amplitude of the perturbations, while for stable equilibria, the amplitude of the perturbations will decay. As seen in Sect. 4, all equilibria have spatially uniform polymer fraction ($\theta _p$) and cell density (*n*) but they need not have spatially uniform film height (*h*). However, in this section we restrict our attention to equilibria with spatially uniform *h*, to facilitate finding analytic solutions and, hence, simplify our analysis.

We denote the dimensionless equilibrium values by asterisks, $L^*$, $\theta ^*$, $n^*$, $h^*$, $v^*$ (where $v^*=0$). The equilibrium values of *h*, *n* and *L* are used as the characteristic values to scale these variables; this means that $h^*=n^* =L^* = 1$. We introduce a short timescale, ${\hat{T}}$, such that $T = \delta {\hat{T}}$ where $\delta \ll 1$, and expand our solutions as power series in $\delta$:

7.1 $$\begin{aligned} L({{\hat{T}}})&= L_{0} + \delta L_{1}({{\hat{T}}}) + \delta ^2 L_{2}({{\hat{T}}}) + ..., \nonumber \\ v_p(X,{{\hat{T}}})&= v_{0}(X) + \delta v_{1}(X,{{\hat{T}}}) + \delta ^2 v_{2}(X,{{\hat{T}}}) + ..., \end{aligned}$$

with expansions for $\theta _p$, *h* and *n* similar to that for $v_p$. We take the spatial perturbations to have an amplitude $\epsilon$, where $\delta \ll \epsilon \ll 1$. (Note that the amplitude $\epsilon$ is distinct from the aspect ratio $\varepsilon$ exploited in Sect. 3.) We then take the series (7.1), etc., and expand each of the terms $L_j$, $v_j$, $\theta _j$, $h_j$ $n_j$, $j=1,2,\ldots$ in powers of $\epsilon$, for example,

$$\begin{aligned} L_j&= L_{j0} + \epsilon L_{j1} + \epsilon ^2L_{j2} + \ldots , \\ v_j&= v_{j0} + \epsilon v_{j1} + \epsilon ^2v_{j2} + \ldots . \end{aligned}$$

We take the initial conditions to be

7.2a $$\begin{aligned} L_0&= 1, \end{aligned}$$

7.2b $$\begin{aligned} v_0&= {v_{00}+}\epsilon v_{01}(X) + \epsilon ^2 v_{02}(X) + ..., \end{aligned}$$

7.2c $$\begin{aligned} \theta _0&= \theta ^* + \epsilon \theta _{01}(X), \end{aligned}$$

7.2d $$\begin{aligned} n_0&= 1 + \epsilon n_{01}(X), \end{aligned}$$

7.2e $$\begin{aligned} h_0&= 1 + \epsilon h_{01}(X). \end{aligned}$$

Note that we set $L_0 = L^* = 1$, i.e. we do not perturb the initial length of the gel from its equilibrium value. Since our initial conditions are equilibria, we have $v_{0 0}=0$, with higher order terms of $v_0$ determined through analysis of the momentum balance equation (6.1d). We set $\theta _{01} = \cos (\gamma X)$, $n_{01} = N_{01} \cos (\gamma X)$, and take $h_{01} = -H_{01} \cos (\gamma X)$, where $N_{01}$ and $H_{01}$ are constants that are ${\mathcal {O}} (1)$. (Given that we expect *h* to change in the opposite way to $\theta _p$ and *n*, we have taken $h_{01}$ to have the opposite sign to that of $n_{01}$ and $\theta _{01}$ without loss of generality.) As required, $\theta _0$, $n_0$ and $h_0$ satisfy the symmetry boundary conditions (6.2a), (6.2b) and (6.2c) at $X=0$ for any choice of $\gamma$. The no-flux boundary conditions (6.2a) and (6.2b) at $X=1$ require that $\gamma = Z \pi$ for some positive integer *Z*. Finally, we wish to ensure that, for any choices of the constants $H_{01}$ and $N_{01}$, the masses of polymer and cells under the perturbed initial conditions are, to ${\mathcal {O}} (\epsilon )$, equal to the masses for the unperturbed initial conditions (which are $\theta ^*$ for the polymer and 1 for the cell density). Integrating $\theta _0 h_0 L_0$ across the spatial domain $0 \le X \le 1$, we have

$$\begin{aligned} \theta ^* = \int _0^1 (\theta _0 h_0 L_0) dX = \theta ^* + \epsilon \frac{1}{\gamma } \sin (\gamma ) - \epsilon \frac{\theta ^* H_{01}}{\gamma } \sin (\gamma ) + {\mathcal {O}}(\epsilon ^2). \end{aligned}$$

Since $\sin (\gamma ) = 0$ for all valid choices of $\gamma$, we see that mass is conserved to ${\mathcal {O}}(\epsilon )$, regardless of our choice of $H_{01}$. On evaluating the integral of $n_0 h_0 L_0$, we find that we are similarly free to set $N_{01}$ to any ${\mathcal {O}}(1)$ value.

To find $v_{01}$, we use equation (6.1d) at ${\mathcal {O}}(\epsilon )$, obtaining the expression

7.3 $$\begin{aligned} \frac{\partial {v_{01}}}{\partial {X}}&= \frac{(1 - \theta ^*)}{2\xi \theta ^*} \frac{\partial {}}{\partial {X}} \left( -\theta ^* f''(\theta ^*) \frac{\partial {\theta _{01}}}{\partial {X}} + \frac{\tau _0}{1+\lambda } \frac{\partial {\theta _{01}}}{\partial {X}} + \theta ^* \frac{2 \tau _0 }{(1+\lambda )^2} \frac{\partial {n_{01}}}{\partial {X}} \right) \nonumber \\&\quad \;\; -\frac{1}{2 {\mathcal {R}}} \left( -\theta ^* f''(\theta ^*) \theta _{01} + \frac{\tau _0}{1+\lambda } \theta _{01} + \theta ^* \frac{2 \tau _0 }{(1+\lambda )^2} n_{01} \right) . \end{aligned}$$

After substituting for $\theta _{01}$ and $n_{01}$, this simplifies to

7.4 $$\begin{aligned} \frac{\partial {v_{01}}}{\partial {X}} = \left( \frac{(1 - \theta ^*)}{2\xi \theta ^*} \gamma ^2 + \frac{1}{2 {\mathcal {R}}} \right) z \cos (\gamma X), \end{aligned}$$

where

7.5 $$\begin{aligned} z = \theta ^* f''(\theta ^*) - \frac{\tau _0 }{1+\lambda } - \theta ^* \frac{2 \tau _0 N_{01} }{(1+\lambda )^2}. \end{aligned}$$

We therefore have the solution

7.6 $$\begin{aligned} v_{01} = \left( \frac{(1 - \theta ^*)}{2\xi \theta ^*} \gamma + \frac{1}{2 {\mathcal {R}}\gamma } \right) z \sin (\gamma X). \end{aligned}$$

Solving the mass conservation equations (6.1b), (6.1c) at ${\mathcal {O}}(\delta )$, we find that $\theta _{10} = n_{10} = h_{10} = 0$, as these terms depend on $v_{00} = 0$. The kinematic boundary condition (6.2e) for *L* similarly gives $L_{10} = 0$.

We therefore consider the mass conservation equations at ${\mathcal {O}}(\delta \epsilon )$. From equation (6.1b), we find

$$\begin{aligned} \frac{\partial {h_{11}}}{\partial {{\hat{T}}}} = \frac{\partial {v_{01}}}{\partial {X}} \quad \Rightarrow \quad h_{11} = \frac{\partial {v_{01}}}{\partial {X}}{\hat{T}}. \end{aligned}$$

Similarly, we find from equations (6.1a) and (6.1c) respectively that

$$\begin{aligned} \theta _{11} = -{2\theta ^*}\frac{\partial {v_{01}}}{\partial {X}}{\hat{T}},\qquad n_{11} = \left( -{2}\frac{\partial {v_{01}}}{\partial {X}} + {D} \frac{\partial ^2 n_{01}}{\partial X^2} \right) {\hat{T}}. \end{aligned}$$

Finally, from the kinematic boundary condition at ${\mathcal {O}} (\delta \epsilon )$, we find that $L_{11} = v_{01}\vert _{X=1} = 0$. Thus, we have the small time analytic solutions

7.7a $$\begin{aligned} \theta _p(X,{\hat{T}})&= \theta ^* + \left[ 1- z \theta ^* \delta {\hat{T}} \left( \frac{(1 - \theta ^*)}{\xi \theta ^*} \gamma ^2 + \frac{1}{{\mathcal {R}}} \right) \right] \epsilon \cos (\gamma X) + {\mathcal {O}}(\delta ^2), \end{aligned}$$

7.7b $$\begin{aligned} n(X,{\hat{T}})&= 1 +\left[ 1 + \delta {\hat{T}} D \gamma ^2 - \frac{z \delta {\hat{T}} }{N_{0 1} } \left( \frac{(1 - \theta ^*)}{\xi \theta ^*} \gamma ^2 + \frac{1}{{\mathcal {R}}} \right) \right] \epsilon N_{01} \cos (\gamma X) + {\mathcal {O}}(\delta ^2), \end{aligned}$$

7.7c $$\begin{aligned} h(X,{\hat{T}})&= 1 - \left[ 1 - \frac{z \delta {\hat{T}}}{2 H_{01}} \left( \frac{(1 - \theta ^*)}{ \xi \theta ^*} \gamma ^2 + \frac{1}{ {\mathcal {R}}} \right) \right] \epsilon H_{01} \cos (\gamma X) + {\mathcal {O}}(\delta ^2), \end{aligned}$$

7.7d $$\begin{aligned} L({\hat{T}})&= 1 + {\mathcal {O}} (\delta ^2) . \end{aligned}$$

We note that these solutions satisfy the no-flux boundary conditions at $X=1$.

The behaviour of the amplitudes of the perturbations can be determined by considering the square-bracketed terms in equations (7.7). We see that the growth or decay of the perturbations of $\theta _p$ and *h* is governed by the sign of *z*. For $z > 0$, the magnitudes of the spatial perturbations are decreasing in time, and accordingly, the local spatial variations decay. For $z < 0$ on the other hand, the amplitudes of the polymer fraction and height perturbations grow, indicating that the equilibrium is unstable. The cell density (7.7b) follows this same behaviour, although the presence of diffusion can be stabilising, depending on the balance of parameters. The stability conditions for spatially uniform equilibria of the thin film model, including the value of *z*, are thus equivalent to those presented in Reoch et al. (2022) for the one-dimensional case; we refer readers to the stability diagrams there given and do not give similar figures here.

## Thin film numerics

We now present our numerical scheme to solve the thin film equations (6.1) in the spatially non-uniform case. We being by re-writing the equations for $\theta _p$ and *h* in conservative form by defining the quantities $Q(X,T) = \theta _ph$ and $W(X,T) = \theta _sh = (1-\theta _p)h$, and noting the following relations:

$$\begin{aligned} h = Q + W, \quad \theta _p= \frac{Q}{Q+W}, \quad \theta _s= \frac{W}{Q+W}. \end{aligned}$$

We then transform the chemical potential $\mu _s$ into a function of *Q* and *W*. Equations (6.1a), (6.1b) and (6.1d) can then be expressed respectively as:

8.1a $$\begin{aligned}&\frac{\partial {Q}}{\partial {T}} - X \frac{\dot{L}}{L} \frac{\partial {Q}}{\partial {X}} + \frac{1}{L} \frac{\partial {}}{\partial {X}} \left( Q v_p \right) = 0,\end{aligned}$$

8.1b $$\begin{aligned}&\frac{\partial {W}}{\partial {T}} - X \frac{\dot{L}}{L} \frac{\partial {W}}{\partial {X}} + \frac{1}{L} \frac{\partial {}}{\partial {X}} \left( W v_p \right) - \frac{2 (Q+W)}{L} \frac{\partial {v_p}}{\partial {X}} = 0, \end{aligned}$$

8.1c $$\begin{aligned}&\frac{2 (Q+W)}{L}\frac{\partial {v_p}}{\partial {X}} - \frac{1}{L^2}\frac{\partial {}}{\partial {X}}\left( V_1(Q, W, n) \right) + V_2(Q, W, n) = 0, \end{aligned}$$

where

8.2a $$\begin{aligned} V_1(Q, W, n)&= \frac{1}{\xi } \left( 2 \chi W -(Q+W) - \frac{W}{N} \left( 1 + \frac{W}{Q} \right) \right) \frac{\partial {}}{\partial {X}} \left( \frac{Q}{Q+W}\right) \nonumber \\&\qquad + \frac{\tau _0}{\xi } \frac{W(Q+W)}{Q} \frac{\partial {}}{\partial {X}} \left( \frac{Q n^2 }{(Q+W)(1+\lambda n^2) } \right) , \end{aligned}$$

8.2b $$\begin{aligned} V_2(Q, W, n)&= \frac{1}{{\mathcal {R}}} \left( \log \left( \frac{W}{W+Q} \right) + \left( 1 - \frac{1}{N} + \frac{\tau _0 n^2}{1+\lambda n^2} \right) \frac{Q}{Q+W} + \chi \left( \frac{Q}{Q+W} \right) ^2 \right) . \end{aligned}$$

The cell advection–diffusion equation remains as given in equation (6.1c). Hence, our numerical code solves the system given by (8.1a) - (8.1c) together with (6.1c).

For this, we implement a finite difference scheme in MATLAB which discretises the system of equations using a uniform spatial grid between $X=0$ and $X=1$. The force balance equation (8.1c) is first-order in velocity $v_p$, and therefore we use a cumulative trapezoidal scheme to numerically integrate the expression across the spatial domain and update the velocity at each new time step. Central differencing is used for spatial derivatives in (8.1c), except for the derivatives of (8.2a) at the endpoints of the domain, where one-sided differences are used. A Crank-Nicolson method is used to solve equations (8.1a), (8.1b) and (6.1c). The end time is chosen to be large enough that the gel reaches a steady state or $\theta _p$ approaches 0 or 1 (in which case our model breaks down).

We find that this scheme conserves mass effectively. Using a time step *dT* between $10^{-6}$ and $10^{-5}$ and spatial step $dX = 0.025$ in the simulations which follow, the worst-case change in mass between initial time and end time for the cell density or polymer fraction was $2.69 \times 10^{-5} \%$. To check our numerical scheme, we compared the full numerical solution with the small time solution detailed in Sect. 7, and also compared the solution obtained with this code for uniform initial conditions with the ODE solution from Sect. 5.1, finding good agreement in each case (results not shown). While our numerical scheme has performed well over a wide range of parameter choices and initial conditions, we note that some instability and non-convergence has been encountered for particular parameter combinations where the gel evolution is rapid, e.g. with large values of $\tau _0$ or $\chi$. We do not consider such cases here.

Throughout the simulations presented in this section, we keep certain parameters fixed and study the effects of changing others between simulations. The fixed parameters and their values are given in Table 1. The values used for the parameters and initial conditions which are varied between examples are presented in Table 2. We note that the initial conditions for $\theta _p$, *n* and *h* will have spatially varying components included on occasion in the simulations which follow; accordingly, we scale the initial conditions for height and cell density using the average initial value for each, such that the mean values ${\bar{h}}_I = \int _0^1 h_I(X) dX = 1$ and ${\bar{n}}_I = \int _0^1 n_I(X) dX = 1$ or 0 depending on the presence or absence of cells. Similarly, when spatial perturbations are added to $\theta _p$, these are such that ${\bar{\theta }}_I = \int _0^1 \theta _I(X) dX$.

**Table 1:** Dimensionless parameter values which remain fixed for all simulations

Term	Symbol	Value used
Polymer chain length	*N*	100
Initial average polymer fraction	${{\bar{\theta }}}_I$	0.6
Polymer bulk viscosity	$\kappa _{p}$	0
Solvent bulk viscosity	$\kappa _{s}$	0
Contact inhibition parameter	$\lambda$	1
Polymer standard free energy	$\mu _{p}^0$	0
Solvent standard free energy	$\mu _{s}^0$	0
Cell traction coefficient	$\tau _0$	1

**Table 2:** Dimensionless parameters which vary between simulations

Term	Symbol	Values
Initial average cell density	${{\bar{n}}}_I$	0, 1
Cell diffusion coefficient	*D*	0, 1
Mixing parameter	$\chi$	0.4, 0.75
Interface resistance	${\mathcal {R}}$	0.4, 1, 4
Drag coefficient	$\xi$	0.2, 1, 4

### Non-uniform initial conditions

We now consider spatially varying initial conditions in one or more of the polymer fraction, cell density and height. We analyse the gel behaviours emerging in different cases, in particular evaluating whether spatial non-uniformities persist or are smoothed out as the gel evolves over time, and whether perturbing different dependent variables results in different impacts on the final outcome.

#### Spatially varying initial polymer, cell-free gel

We first consider a cell-free gel with a non-uniform initial polymer distribution. We take the initial condition $\theta _I = 0.6 + 0.02 \cos (\pi X)$, noting that $\theta _I$ satisfies the boundary conditions $\partial \theta _p / \partial X = 0$ at $X=0, \, 1$, and that the initial mass of polymer in the gel remains 0.6 under this initial condition. This resembles a gel where the polymer fraction is initially slightly larger around the centre of the thin film at $X=0$. We will firstly discuss the equilibrium outcomes for the gel, then describe how the variables change over time.

**Fig. 4:**
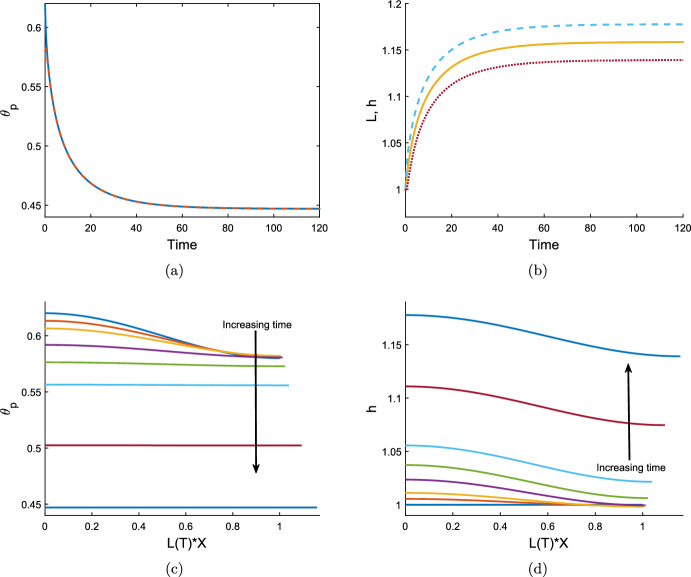
Time evolution of a cell-free gel with non-uniform initial polymer fraction $\theta _I = 0.6 + 0.02 \cos (\pi X)$, and parameters $n_I = 0$, $h_I = 1$, $\chi = 0.75$, $\xi = 1$, ${\mathcal {R}}= 1$, expanding to equilibrium (${\theta }^*, {\bar{h}}^*, L^*) = (0.45, 1.16, 1.16)$. Figure 4a shows $\theta _p$ versus time *T* at $X=0$ (solid blue curve) and $X=1$ (dashed red curve); the curves are identical to graphical accuracy. Figure 4b shows *h* versus *T* at $X=0$ (dashed light blue curve) and $X=1$ (dotted maroon curve), along with *L*(*T*) as the solid gold curve. Figure 4c and d show curves $\theta _p$ versus *X* and *h* versus *X*, respectively, at times $T=0, 0.1, 0.2, 0.5, 1, 2, 8, 120$, with time increasing in the direction of the black arrow. The polymer fraction evens out to a uniform equilibrium as the gel swells (Fig. 4a and c). Spatial variations develop in the height in response to the initial non-uniform polymer distribution and persist to equilibrium (Fig. 4b and d)

We see in this environment that spatially varying steady states can be found without the presence of cells. Figure 4a displays the time evolution of $\theta _p(X,T)$ at $X=0, \, 1$, while Fig. 4b shows the time evolution of *h*(*X*, *T*) at $X=0, \, 1$ as well as *L*(*T*). We see that the gel height *h* evolves to a non-uniform equilibrium here. The mixing parameter $\chi = 0.75$ is at a value that promotes mixing between the polymer and solvent. This drives an osmotic pressure gradient, causing the gel to swell to an equilibrium state, with solvent flowing into the gel. The polymer fraction converges to a spatially uniform value as it evolves, reaching a steady state where $\theta ^* = 0.45$. Meanwhile, we see that non-uniformities develop in the gel height; these spatial variations persist at equilibrium where the mean equilibrium value ${\bar{h}}^* = 1.16$ and the amplitude $A_{h^*} = 0.019$, where $A_{h^*} = (h^*_{\text {max}} - h^*_{\text {min}})/2$. The amplitude $A_{h^*}$ is of a similar magnitude to the amplitude of the initial polymer fraction. The gel length at equilibrium $L^* = 1.16$ is equal to the mean equilibrium value of the height ${\bar{h}}^*$.

Figures 4c and d show the spatial distributions of $\theta _p$ and *h* respectively across the gel length at increasing points in time. As seen in Fig. 4c, the initial non-uniformity in the polymer distribution quickly smooths out so that $\theta _p$ is uniform across the spatial domain. Meanwhile, Fig. 4d demonstrates that sinusoidal variations matching the shape of those in the initial polymer arise in the gel height. These variations persist over time, resulting in varying height at the gel’s steady state.

In response to the osmotic pressure gradient here driven by the free energy, we see more solvent enter the gel over early time (e.g. $T=0$ to $T=0.5$) in the regions of higher polymer fractions near $X=0$, resulting in these areas of the gel becoming locally thicker. This is seen in the gel height increasing to a greater degree close to $X=0$ since more solvent is entering the gel in that region. Conversely, we see $\theta _p$ increase near $X=1$ over this time period, i.e. there is some localised contraction in the gel due to the initial presence of more solvent in this region. This corresponds to decreases in *h* seen at corresponding times. By $T=1$, the gel swells across the spatial domain, with a uniform polymer profile developing as solvent continues to enter the gel more rapidly in areas of greater polymer concentration. The height continues to increase as the gel swells, maintaining its non-uniform distribution. These variations that develop and persist in *h* correspond to local variations in mass across the spatial domain that exist from the initial non-uniformity in $\theta _I$. Accordingly, while the fraction of polymer is constant by the time the gel equilibrates, the mass of polymer per unit length $\theta ^* h^*(X)$ varies in space.

In Sect. 4, we found that $\partial \theta _p/ \partial X = 0$ is a necessary condition for equilibrium in the thin film. We see here that the polymer is redistributed evenly such that there is a balance in chemical potential from gel to solvent and within the gel itself. The extra mass at different points in space (coming from the spatially varying height) enables a constant polymer fraction to be maintained at equilibrium. We note that this model does not consider surface tension. With surface tension present, we might expect the variations in height to smooth over time as well; however, in its absence, there is no force driving the surface to flatten out and we see the non-uniformities persist at equilibrium.

We see the same qualitative outcome for the gel when taking non-uniform $h_I$ with uniform $\theta _I$, i.e. at the resulting steady state, $h^*$ will vary in space while $\theta ^*$ is uniform (results not shown). As seen in the previous example, the variations in mass across the spatial domain allow for a uniform polymer fraction to be maintained at equilibrium.

#### Spatially varying initial polymer, cell-gel system

**Fig. 5:**
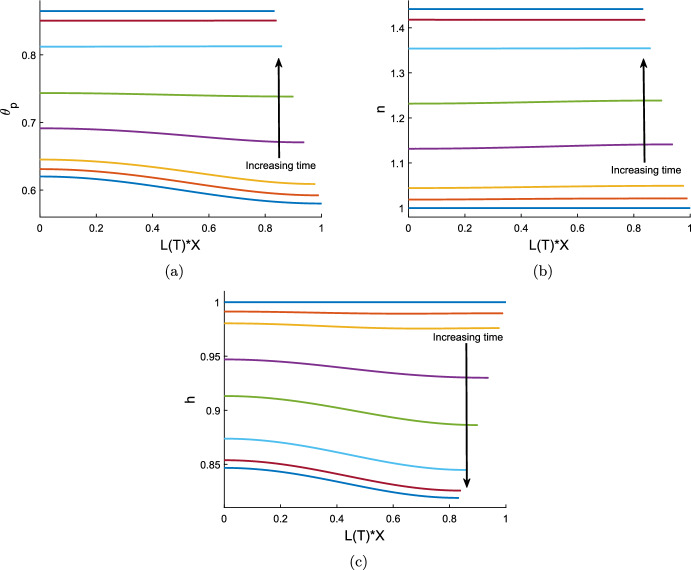
Time evolution of a cell-gel system with non-uniform initial polymer fraction $\theta _I = 0.6 + 0.02 \cos (\pi X)$, and parameters $n_I = 1$, $h_I = 1$, $\chi = 0.75$, $\xi = 1$, ${\mathcal {R}}= 1$, $\tau _0=1$, $D=1$, contracting to equilibrium (${\theta }^*, n^*, {\bar{h}}^*, L^*) = (0.86, 1.44, 0.83, 0.83)$. Figure 5a shows that spatial variations in the polymer profile decay quickly over time, whilst in Fig. 5b small spatial variations briefly emerge in the cell density, but dissipate before the gel equilibrates. By contrast, in Fig. 5c spatial variations which emerge in the gel height increase in magnitude throughout to equilibrium. Profiles are plotted at $T=0, 0.1, 0.2, 0.5, 0.8, 1.2, 1.6, 3$, with time increasing in the direction of the black arrow

We now take the system presented in Sect. 8.1.1 and introduce a cell population where $n_I = 1$ and $\tau _0 = 1$. We maintain the non-uniform initial condition for polymer, ${\theta _I} = 0.6 + 0.02 \cos (\pi X)$. Figure 5 displays this system’s evolution. While the gel was previously seen to swell, the introduction of cells switches the gel’s behaviour to contraction, with the forces the cells generate outweighing the chemical potential gradient. The gel reaches a steady state where $\theta ^* = 0.86$, $n^* = 1.44$, ${\bar{h}}^* = 0.83$, $L^* = 0.83$. We note that this is the same equilibrium in $\theta ^*$ and $n^*$ as obtained using the same parameter values in our one-dimensional model (Reoch et al. 2022).

As in Sect. 8.1.1, the non-uniformity in $\theta _p$ evens out over time, with *h* developing spatial variations that remain present at equilibrium; Fig. 5a and c show how the spatial profiles of $\theta _p$ and *h* respectively change over time. Meanwhile, variations also appear in the cell density *n* while the gel contracts; the cell density increases more around $X=1$ in response to the smaller initial polymer fraction there. However, as time progresses, the variation in *n* decays due to the presence of diffusion (see Fig. 5b).

#### Spatially varying initial cell density, cell-gel system

**Fig. 6:**
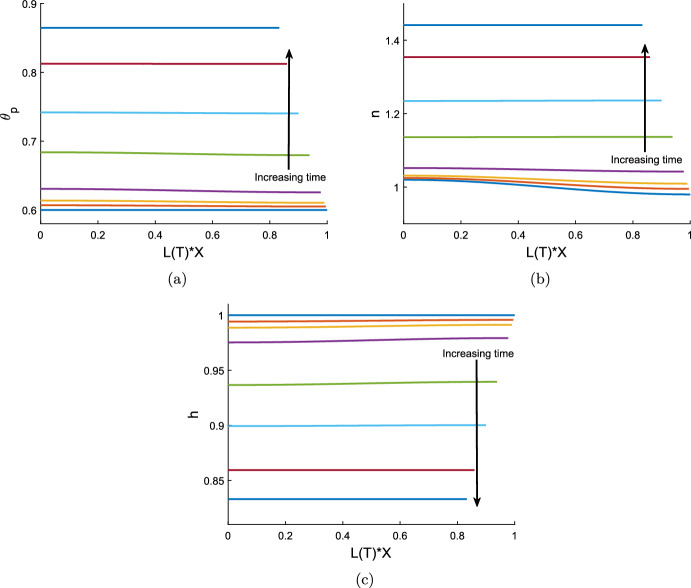
Time evolution of a cell-gel system with non-uniform initial cell density $n_I = 1 + 0.02 \cos (\pi X)$, and parameters $\theta _I = 0.6$, $h_I = 1$, $\chi = 0.75$, $\xi = 1$, ${\mathcal {R}}= 1$, $\tau _0=1$, $D=1$, contracting to equilibrium (${\theta }^*, n^*, {\bar{h}}^*, L^*) = (0.86, 1.44, 0.83, 0.83)$. Spatial variations briefly emerge in the polymer fraction (Fig. 6a) and the gel height (Fig. 6c), which dissipate before the gel equilibrates, whilst spatial variations in the cell density decay quickly over time (Fig. 6b). Profiles are plotted at $T=0, 0.06, 0.1, 0.2, 0.5, 0.8, 1.2, 3$, with time increasing in the direction of the black arrow

We now take the initial cell density to be spatially varying, such that $n_I = 1 + 0.02 \cos (\pi X)$, with a uniform initial polymer fraction $\theta _I = 0.6$. Small spatial variations arise in both the polymer fraction and height here as the gel evolves (see Fig. 6). The non-uniform cell distribution, shown in Fig. 6b, leads to greater forces initially being applied in the negative *X*-direction; this cell traction induces spatial gradients in the polymer profile and, accordingly, the height, as more solvent is forced from the gel. Figure 6a shows $\theta _p$ increasing towards $X=0$ over early time in response to the cell force gradient, while in Fig. 6c, we see a corresponding decrease in height around $X=0$. Contrary to the previous examples seen in this Section, the non-uniformity that arises in the height as the gel evolves does not continue to equilibrium. In this instance, with the polymer initially uniform, the small local variations in the gel’s thickness do not persist at equilibrium. As required, the cell density is constant when the gel equilibrates, with the strong diffusion coefficient playing a significant role in smoothing out the initial variations. The gel reaches the same steady state as the previous example with $\theta _p^* = 0.86$, $n^* = 1.44$, $h^* = L^* = 0.83$; therefore, we see that varying the initial cell distribution does not led to greater contraction in the gel.

#### Spatially varying initial height, cell-gel system

We now vary the initial height for this gel, such that $h_I = 1 + 0.02 \cos (\pi X)$, while taking $\theta _I = 0.6$ and $n_I = 1$ to be constant. The gel again contracts to an equilibrium with $\theta _p$ and *n* being spatially uniform, while spatial variations in *h* persist through to the gel’s steady state. The mean equilibrium values of the model variables are unchanged from previous examples. As this example does not demonstrate any new qualitative outcomes, we do not include any figures.

We note that taking different combinations of spatially varying initial conditions in *h*, *n* and $\theta _p$ will result in the same qualitative outcomes for the system – if $\theta _p$ or *h* are spatially varying initially, then *h* will be spatially dependent at equilibrium, regardless of the initial cell density; only varying *n* initially will not induce a non-uniform equilibrium by itself.

### Influence of drag and resistance

**Fig. 7:**
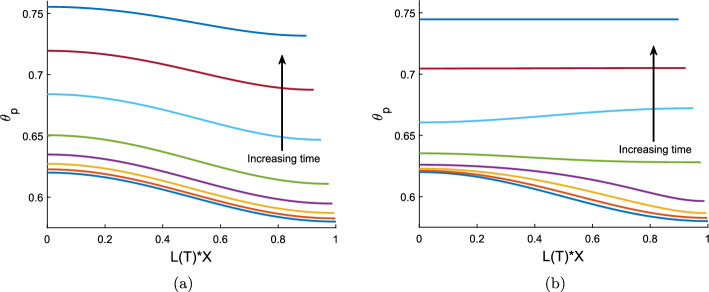
The effect of varying the drag parameter, $\xi$. For large drag ($\xi = 4$, Fig. 7a) spatial variations in polymer density slowly recede as the gel contracts over time. By contrast, in the case of low drag ($\xi = 0.2$, Fig. 7b), the spatial variations quickly smooth out as the gel contracts. Profiles are plotted at $T=0, 0.02, 0.05, 0.1, 0.2, 0.4, 0.6, 0.8$, with time increasing in the direction of the black arrow. Initial conditions and parameter values otherwise as given in Fig. 5

We now investigate the effect that the drag parameter $\xi$ and the resistance parameter ${\mathcal {R}}$ have on the gel’s evolution. We take a contracting gel with cells, with parameter values and initial conditions as given in Fig. 5, where the initial polymer fraction is non-uniform. We reiterate that in this case, the gel will equilibrate to a uniform value of polymer, $\theta ^* = 0.86$. We now modify the drag parameter $\xi$. In Fig. 7a and b we compare the polymer fraction’s spatial evolution over early time (up to $T=0.8$) for high drag ($\xi = 4$) and low drag ($\xi = 0.2$) respectively. In the high drag case, we see little change over this time to the spatial structure of the gel as it contracts. The amplitude of the variations in the polymer fraction decreases over time (amplitude ${\mathcal {A}}_{\theta _p} = 0.007$ at $T=0.8$); however, it retains the sinusoidal shape of the initial condition. In this case, the large drag coefficient slows down solvent flow through the gel in the *x*-direction. It is therefore easier for solvent to flow out of the gel primarily in the thin direction. It does this at a relatively uniform rate across the domain, hence the spatial distribution only slowly decreases in amplitude. With low drag on the other hand, we see that the polymer fraction changes more rapidly near $X=1$ than in the high drag case. The polymer moves quickly towards a uniform spatial distribution, since it is now easier for fluid to flow longitudinally within the gel as well as in the vertical direction. With more cell forces being applied in the area near $X=1$, the polymer fraction increases at one point (at around $T=0.4$) as it evolves, before evening out again as the gel moves towards its equilibrium state. We note that the polymer fraction reaches a uniform steady state at $T \approx 3$ in both the low and high drag cases here (results not shown).

**Fig. 8:**
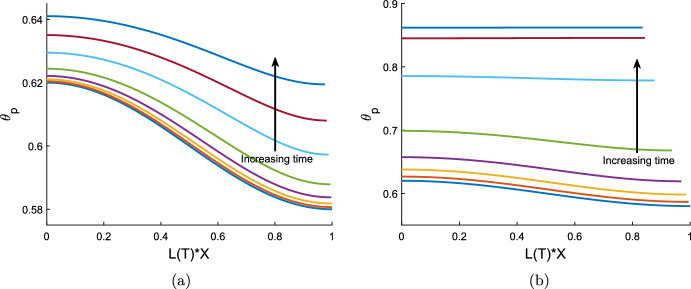
The effect of varying the resistance parameter, ${\mathcal {R}}$. When resistance is high (${\mathcal {R}}= 4$, Fig. 8a) the polymer fraction decreases very slowly due to the impermeability of the boundary, whilst when the boundary is more permeable (${\mathcal {R}}= 0.4$, Fig. 8b), contraction is much faster. Profiles are plotted at $T=0, 0.02, 0.05, 0.1, 0.2, 0.4, 0.6, 0.8$, with time increasing in the direction of the black arrow. Initial conditions and parameter values otherwise as given in Fig. 5

The resistance parameter ${\mathcal {R}}$ affects the speed at which fluid can flow across the gel-solvent interface at both $y=h$ and $X=1$. We see dramatic differences when comparing the evolution in $\theta _p$ for low resistance with ${\mathcal {R}}= 0.4$ in Fig. 8b with high resistance, ${\mathcal {R}}= 4$ in Fig. 8a (we note that in both these examples $\xi =1$). For low resistance, the gel quickly contracts, with $\theta _p$ smoothing out as it moves towards its steady state. With this small value of ${\mathcal {R}}$, the gel has equilibrated by $T=0.8$. For high resistance, the evolution is significantly slower. Indeed, by $T=0.8$, the polymer fraction has only increased to approximately $\theta _p= 0.64$ at its maximum. Interestingly, in this case, while the fraction of polymer is only changing slowly, the amplitude has halved from its initial value by $T=0.8$. This indicates that, while the resistance is slowing the flow of solvent across the gel’s boundary, solvent inside the gel is flowing quickly enough to flatten the polymer’s distribution.

### Zero-diffusion case

The derivation of the thin film model, wherein the diffusive flux terms are used to derive that *n* is independent of *y*, requires *D* to be ${\mathcal {O}}(1)$. Accordingly, the examples presented in this chapter so far have been generated with $D=1$. Nevertheless, for the sake of completeness, we now investigate the outcomes for the system without diffusion, although we cannot guarantee the validity of our thin film reduction in this case. With zero diffusion, cells must move with the polymer and the cell distribution does not need to be uniform at steady state, indicating that $\theta _p$ in turn is also not required to be uniform at equilibrium.

**Fig. 9:**
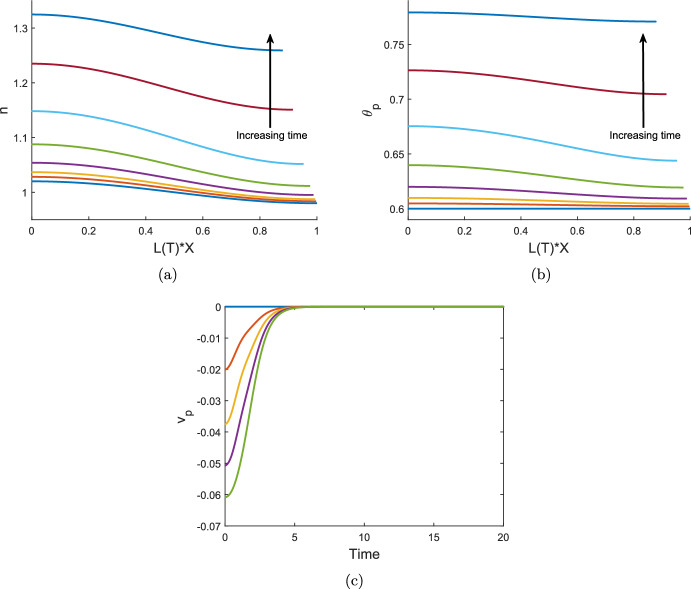
Time evolution of a cell-gel system with zero cell diffusion, a non-uniform initial cell density $n_I = 1 + 0.02 \cos (\pi X)$, and parameters $\theta _I = 0.6$, $h_I = 1$, $\chi = 0.4$, $\xi = 1$, ${\mathcal {R}}= 1$, $\tau _0=1$, $D=0$, contracting to equilibrium (${\bar{\theta }}^*, {\bar{n}}^*, {\bar{h}}^*, L^*) = (0.78, 1.29, 0.88, 0.88)$. Spatial variations in the cell profile grow and persist at equilibrium (Fig. 9a), whilst similar spatial variations in the polymer density also emerge and persist (Fig. 9b); profiles are plotted at $T=0, 0.05, 0.1, 0.2, 0.4, 0.8, 1.6, 8$, with time increasing in the direction of the black arrow. Figure 9c shows curves of the velocity $v_p$, versus time *T*, at $X=0$ (blue), $X=0.25$ (red), $X=0.5$ (yellow), $X=0.75$ (purple), $X=1$ (green); $v_p$ goes to zero across the spatial domain as the gel reaches its spatially varying equilibrium

By way of example, we take $D=0$ with initial conditions $n_I = 1 + 0.02 \cos (\pi X)$, $\theta _I = 0.6$, $h_I = 1$. We note here that due to issues with code convergence, we have run these examples with a smaller interaction energy than previously, taking $\chi = 0.4$. We show in Fig. 9a, b that with no diffusion and a non-uniform initial cell density, we obtain equilibria that are non-uniform in the cell density and polymer fraction. This reflects similar examples in our 1D model (Reoch et al. 2022). In this instance, the gel reaches a mean equilibrium cell density ${\bar{n}}^* = 1.29$ with amplitude ${\mathcal {A}}_{n^*} = 0.033$, which is greater than the initial amplitude. The polymer fraction evolves to mean equilibrium value ${\bar{\theta }}^* = 0.78$ with amplitude ${\mathcal {A}}_{\theta ^*} = 0.004$, while for the height, ${\bar{h}}^* = 0.88$ with amplitude ${\mathcal {A}}_{h^*} = 0.002$. Figure 9c demonstrates the time evolution of velocity $v_p$ at different points in the spatial domain. We see that the velocity goes to zero at all shown spatial points, confirming that the gel is at a steady state. With only cells taken to be initially non-uniform, the gel reaches a steady state where the cells, polymer and height are all non-uniform. This is a significant difference to the case with diffusion, where non-uniform initial cell profiles did not lead to spatially varying steady states. While this simulation does not fit with the particular derivation of the thin film system here, it does demonstrate the possibility that spatially dependent solutions may occur in both the polymer and cells. Examples with a small diffusion coefficient (e.g. $D \approx 1\text {x}10^{-3} - 1\text {x}10^{-5}$) were found to reach a similar non-uniform quasi-steady state; however, given the presence of the small diffusive flux, cells and polymer moved very slowly towards a uniform distribution (results not shown).

## Discussion

In this paper, we have presented a model to study the behaviour of a thin film of gel floating freely within a bath of solvent. Starting from the equations of our earlier model of cell-induced gel contraction (Reoch et al. 2022), we exploited the small aspect ratio $\varepsilon$ of the problem, assumed symmetry about $x=0$ and $y=0$, and showed that under a particular set of scalings, the original two-dimensional system of equations can be reduced to a one-dimensional system of four coupled PDEs. This leading-order model consists of equations for the gel half-height *h* (3.21), polymer fraction $\theta _p$ (3.22), cell density *n* (3.28) and polymer velocity $v_p$ (3.41). Such a model, considering cell traction stresses and osmotic pressure in this thin film setting has not, to our knowledge, been presented previously.

The thin film model presented here has some key differences to the 1D model presented in Reoch et al. (2022). In the previous geometry, all solvent flowed in or out of the gel horizontally through the gel’s endpoints at $X=\pm 1$. In contrast, in the thin film model, most of the solvent flow into or out of the gel occurs across the long boundaries at $y=\pm h$. This manifests itself in the additional terms seen in equation (3.20), which describes (at leading order) mass conservation of the solvent fraction across the thickness of the gel, and includes a source term which depends on the balance of chemical and cell potentials, scaled by the resistance of the interface to fluid flow. This source term effectively emerges as a result of integrating over the height of the gel. Another key difference is that we have an equation (3.41) for the polymer velocity in the thin film model which is first order in space, as opposed to a second order equation previously. Finally, we note that the thin film approximation relies on the presence of the cell diffusion terms to show that $n_0$ is independent of *y*; therefore, unlike the previous 1D case, we cannot set $D=0$ without violating one of the assumptions underpinning the thin film model. Despite these differences in the detail of the equations, the conditions for a steady state remain the same as in the one-dimensional model of Reoch et al. (2022).

For spatially uniform initial conditions, we have shown that the solution of thin-film equations is also spatially uniform in the dependent variables $\theta _p$, *n* and *h*. In fact, the model can be reduced to a single ODE in time for the height *h*. By contrast, in the 1D Cartesian model such spatially-uniform solutions only occur for no drag, $\xi =0$ (Keener et al. 2011b; Reoch et al. 2022). This is because, in the 1D case, fluid must flow into or out of the gel through the boundary at $X=1$, producing non-uniform spatial profiles in the dependent variables as it moves through the gel (these non-uniformities generally smooth out by the time the gel equilibrates). In the thin film, solvent can flow across the long boundary at $y=h$ and so drag has less influence on the gel’s behaviour. The dynamics of the ODE model are driven by the balance between cell and chemical potentials internally with the external chemical potential. As expected, given the same equilibrium conditions exist for both 1D and thin film models, the simulations reach the same equilibrium values for $\theta ^*$ and $n^*$ given the same parameter values and initial conditions. Interestingly, in deriving the thin film ODE model, we find that the scaled thin film length and height are equal. This relationship was assumed in an *ad hoc* manner in the work of Stevenson et al. (2010) when calculating cell traction. Our model thus provides a validation for their assumption in the case of uniform initial conditions.

For non-uniform initial conditions, we have derived small-time solutions, allowing for predictions of the stability of steady states. We also performed numerical simulations which show more complex behaviour compared to the spatially uniform case. The behaviour of the gel is still primarily driven by flow in the thin direction; however, the terms involving drag and spatial derivatives in the dependent variables are now active. Accordingly, we see spatial variations arising in $\theta _p$, *n* and *h* as the gel evolves. We have found that solutions exist where the gel height is non-uniform at steady state, even though the polymer fraction and cell density are spatially constant. When the initial polymer fraction $\theta _I$ is non-uniform, local variations in the polymer induce solvent flow into or out of the gel to even out the polymer fraction; this correlates with spatial variations in the gel height at these points, reflecting the variations in mass. Similarly, when the initial gel height, $h_I$, is non-uniform, then *h* will be spatially-varying to ensure $\theta _p$ remains constant.

A significant difference in the thin geometry from the 1D case is that increasing drag now reduces spatial changes in the velocity. This is evident from equation (6.1d), where we see that increasing $\xi$ decreases the influence of the spatial derivative terms on the velocity. A consequence of this is evident in Fig. 7a, which shows that, with large drag, initial spatial variations persist longer through the gel’s evolution, as it is easier for fluid to flow vertically out of the gel than across the spatial domain due to the shearing forces present with a large drag coefficient. In the 1D case, we saw that increasing the drag coefficient tended to induce greater spatial gradients in the polymer and cells, as solvent could only enter and leave the gel through the endpoint at $X=1$, and so had to flow across the entire spatial domain. We believe that this may explain why, for the thin film geometry, we have not observed oscillation between swelling and contraction, which we previously observed in the 1D geometry (Reoch et al. 2022).

We have noted that our thin film model derivation assumes that the non-dimensional diffusion coefficient *D* is ${\mathcal {O}}(1)$. Under this assumption, as seen in Sect. 4, we cannot find equilibria in the thin film system with non-uniform polymer or cell distributions. Diffusion causes the cells to spread until a uniform cell density is reached across the gel. We note that examples with diffusion $D \ne 0$ in the 1D case also resulted in uniform equilibria. We have presented simulations with zero diffusion in the thin film here, finding examples where non-uniform equilibria persist in $\theta _p$ and *n*. While we cannot definitively say that $n=n(x,t)$ for the zero diffusion case, we have shown that, if such *y*-independent solutions can exist for $D=0$, then we can find non-uniform equilibria in the thin film environment as well. We note that with small diffusion ($D \approx 0.0001$), we do see quasi-steady states in $\theta _p$ and *n*, where non-uniform states are found which slowly drift towards uniformity as a result of diffusive flux.

Unlike the studies by Trinschek et al. (2016, 2017) discussed in Sect. 1, we do not see situations where, at a certain point, the gel continues expanding lengthwise while its vertical swelling has stopped. Indeed, throughout these simulations, we see that the scaled height (or its mean value) is equal to the scaled gel length at equilibrium. As noted above, this supports modelling assumptions used in Stevenson et al. (2010), although we do note that, in the spatially non-uniform case, the average gel height ${\bar{h}}$ does not equal *L* for all time. In our model, there is no mechanism to cause a change in behaviour and decouple the lengthwise expansion from the vertical movement of the gel. This could possibly occur if the domain was somehow vertically or horizontally limited, or if an additional external pressure was imposed from one particular direction. A different cell force function might also see more emphasis on horizontal forces. An avenue for future work may be to explore whether these dynamics can be factored in, allowing for the expansion or contraction of a gel to be tailored in a certain direction, not just a situation where the length and height evolve similarly as seen here.

There is significant scope to extend this research and validate the model’s behaviours through experimental collaboration. This would allow for parameter values to be fitted and suggest particular regions in the parameter space for deeper analysis to be carried out. The numerical results in this paper suggest further avenues to investigate experimentally, for example, confirming whether gels which are initially uniform in space retain this uniformity as they evolve, and whether small variations in the initial polymer profile do indeed result in spatially varying height profiles.

A number of modelling extensions are also of interest. Inclusion of surface tension would enable a study of its effect in cases where the gel height is found to vary; the aim would be to establish whether surface tension is sufficiently large to smooth out the height in such cases. Typical experimental setups, suggest future consideration of a thin gel on a solid substrate; this removes the symmetry about $y=0$ so making the modelling more complex. Further, there may be cases where mechanical changes in the gel occur on a timescale similar to that of cell proliferation and death, making this an interesting scenario to explore. Again, this would add complexity to the model and its solution, requiring addition of suitable source terms to the PDE for cell density, and we have left this to future work.

## Data Availability

Data sharing not applicable to this article as no datasets were generated or analysed during the current study.

## Nomenclature

**Table:**

Symbol	Description	Symbol	Description
	*Domains*		*Independent variables*
$\Omega _g$	Gel domain	*t*	Time
$\Omega _s$	Pure solvent domain	*x*	Distance along the gel from its centre
$\Omega$	Total domain $\Omega _g\cup \Omega _s$	*y*	Height above the centre of the gel
	*Dependent variables*		*Physical parameters & functions*
$\varvec{e}_p$	Rate-of-strain tensor in polymer phase	*D*	Diffusivity of cells in gel
$\varvec{e}_s$	Rate-of-strain tensor in solvent phase	*f*	Free energy per unit volume of gel
*h*	Gel half-height	*G*	Cell force potential
*L*	Gel half-length	${\mathcal {H}}$	Initial average gel height
*n*	Cell density or number per unit volume	$k_B$	Boltzmann constant
$\varvec{{\hat{n}}}$	Unit normal to gel surface	${\mathcal {L}}$	Initial gel half-length
*P*	Pressure within the gel	*N*	Polymer chain length
$P^e$	Pressure external to the gel	${\mathcal {N}}$	Initial average cell density
$P_{\Delta }$	$P-P^e$	${\mathcal {R}}$	Resistance of boundary to solvent flow
$\varvec{v_p}$	Velocity in the polymer phase	${\mathcal {T}}$	Temperature
$\varvec{v_s}$	Velocity in the solvent phase	$\epsilon$	Initial average gel height over length
$v_p$	*x*-component of polymer velocity $\varvec{v_p}$	$\eta _p$	Dynamic viscosity of polymer phase
$v_s$	*x*-component of solvent velocity $\varvec{v_s}$	$\eta _s$	Dynamic viscosity of solvent phase
$w_p$	*y*-component of polymer velocity $\varvec{v_p}$	$\kappa _p$	Bulk viscosity of polymer phase
$w_s$	*y*-component of solvent velocity $\varvec{v_s}$	$\kappa _s$	Bulk viscosity of solvent phase
$\theta _p$	Volume fraction of polymer	$\lambda$	Contact inhibition parameter
$\theta _s$	Volume fraction of solvent	$\mu _p$	Chemical potential for the polymer
$\Pi$	$P-\theta _pG$	$\mu _s$	Chemical potential for the solvent
$\varvec{\sigma }_p$	Stress tensor in polymer phase	$\mu _s^e$	Chemical potential in external solvent
$\varvec{\sigma }_s$	Stress tensor in solvent phase	$\mu _s^0$	Standard free energy in pure solvent
$\varvec{\sigma }_s^e$	Stress tensor in external solvent	$\mu _{\Delta }$	$\mu _s-\mu _s^e$
${{\bar{\theta }}}, {{\bar{n}}}, {{\bar{h}}}$	Spatially averaged $\theta , n, h$	$\nu _m$	Characteristic volume of a monomer
${{\bar{\theta }}}_I, {{\bar{n}}}_I, {{\bar{h}}}_I$	Initial ${{\bar{\theta }}}, {{\bar{n}}}, {{\bar{h}}}$	$\xi$	Drag coefficient
$\theta ^*, n^*, h^*, L^*$	Equilibria of $\theta , n, h, L$	$\tau _0$	Cell traction strength
${{\bar{\theta }}}^*, {{\bar{n}}}^*, {{\bar{h}}}^*$	Spatially averaged $\theta ^*, n^*, h^*$	$\chi$	Flory interaction parameter

## Thin film approximation: the *y*-independence of the first-order correction terms

In this appendix, we demonstrate that the ${\mathcal {O}}(\varepsilon )$ terms in cell density $n_1$, pressure $P_1$, polymer fraction $\theta _{p_1}$, and polymer axial velocity $v_{p_1}$ are also independent of *y*. This simplifies the derivation of an equation for the leading-order velocity (see Sect. 3.3).

The result follows from essentially the same arguments as used in Sect. 3.1. We begin by integrating equation (2.19) at ${\mathcal {O}} ( \varepsilon ^{-1})$ and using the no-flux boundary condition $\partial n_1 / \partial y = 0$ at $y=h_0$, we find that $n_1 = n_1(x,t)$. This indicates that the first order correction to the cell force function is also independent of *y*, that is, $G_1 = G_1(x,t)$. Evaluating (2.22b) at ${\mathcal {O}}( \varepsilon ^{-1})$ yields

B.1 $$\begin{aligned} \frac{\partial {\Pi _1}}{\partial {y}} = 0, \quad \implies \quad \Pi _1 = \Pi _1(x,t), \end{aligned}$$

where $\Pi _1 = P_1 - \theta _{p_0} G_1 - \theta _{p_1} G_0$. Using (2.25), we find that

B.2 $$\begin{aligned} P_1 - \theta _{p_0} G_1 - \theta _{p_1} G_0 = 0 \end{aligned}$$

at $y=h_0$, and hence $\Pi _1 = 0$. Therefore,

B.3 $$\begin{aligned} P_1 = \theta _{p_0} G_1 + \theta _{p_1} G_0 \end{aligned}$$

everywhere in the domain. Now, equation (2.21b) at ${\mathcal {O}} (\varepsilon )$ becomes

B.4 $$\begin{aligned} \theta _{s_0} \frac{\partial {}}{\partial {y}} \left( \mu _{s_1} + G_0 \theta _{p_1} \right) = 0, \end{aligned}$$

where $\mu _{s_1}$ is the first order correction to $\mu _s$; therefore,

B.5 $$\begin{aligned} \theta _{s_0} \left( \frac{\partial {\mu _{s_1}}}{\partial {\theta _p}} + G_0 \right) \frac{\partial {\theta _{p_1}}}{\partial {y}} = 0. \end{aligned}$$

As in the leading order case, either possible solution to this equation requires that $\theta _{p_1} = \theta _{p_1}(x,t)$; hence $\mu _{s_1}$ must also be independent of *y*. We also, therefore, have that $P_1 = P_1(x,t)$ from equation (B.3).

Evaluating equation (2.22a) at ${\mathcal {O}} (\varepsilon )$, we find

B.6 $$\begin{aligned} -\frac{\partial {\Pi _1}}{\partial {x}} + \frac{\partial {}}{\partial {y}} \left( \theta _{p_0}\frac{\partial {v_{p_1}}}{\partial {y}} + \theta _{p_1} \frac{\partial {v_{p_0}}}{\partial {y}} \right) = 0. \end{aligned}$$

This simplifies to the condition

B.7 $$\begin{aligned} \frac{\partial {}}{\partial {y}} \left( \theta _{p_0}\frac{\partial {v_{p_1}}}{\partial {y}} \right) = 0, \end{aligned}$$

from which, after integrating with respect to *y* and applying interface condition (2.24), we have

B.8 $$\begin{aligned} \theta _{p_0}\frac{\partial {v_{p_1}}}{\partial {y}} = 0, \quad \implies \quad v_{p_1} = v_{p_1}(x,t). \end{aligned}$$

From (2.21b) at ${\mathcal {O}} (\varepsilon )$, we find that

B.9 $$\begin{aligned} v_{s_1} = v_{p_1} - \frac{1}{\xi \theta _{p_1}} \frac{\partial {}}{\partial {x}} \left( \mu _{s_1} + \theta _{p_0}G_1 + \theta _{p_1} G_0 \right) . \end{aligned}$$

Thus, the solvent axial velocity must also be independent of *y* at first order, i.e. $v_{s_1} = v_{s_1}(x,t)$.
